# Hypoxia-induced miR-5100 promotes exosome-mediated activation of cancer-associated fibroblasts and metastasis of head and neck squamous cell carcinoma

**DOI:** 10.1038/s41419-024-06587-9

**Published:** 2024-03-14

**Authors:** Yuansheng Duan, Mengqian Zhou, Beibei Ye, Kai Yue, Feng Qiao, Yuxuan Wang, Qingchuan Lai, Yue Wu, Jiayan Cao, Yansheng Wu, Xudong Wang, Chao Jing

**Affiliations:** 1 Department of Maxillofacial and Otorhinolaryngological Oncology, Tianjin Medical University Cancer Institute & Hospital, National Clinical Research Center for Cancer, Tianjin’s Clinical Research Center for Cancer, Key Laboratory of Basic and Translational Medicine on Head & Neck Cancer, Tianjin, Key Laboratory of Cancer Prevention and Therapy, Tianjin, Tianjin, 300060 China; 2 Department of Thyroid and Breast Surgery, The Second Hospital of Anhui Medical University, Anhui, 230601 China; 3 Department of Oral and Maxillofacial Surgery, School and Hospital of Stomatology, Tianjin Medical University, Tianjin, 300070 China

**Keywords:** Head and neck cancer, Metastasis, Diagnostic markers, Cancer microenvironment

## Abstract

The invasion-metastasis cascade in head and neck squamous cell carcinoma (HNSCC) is predominantly caused by the interaction between tumor cells and tumor microenvironment, including hypoxia as well as stromal cells. However, the mechanism of hypoxia-activated tumor-stroma crosstalk in HNSCC metastasis remains to be deciphered. Here, we demonstrated that HIF1α was upregulated in HNSCC specimens compared with adjacent normal tissues, whose overexpression was associated with lymph node metastasis and predicted unfavorable prognosis. HIF1α expression correlated positively with the levels of miR-5100 as well as α-SMA, the marker of CAFs. Hypoxia/HIF1α regulated transcriptionally miR-5100 to promote the degradation of its target gene QKI, which acts as a tumor suppressor in HNSCC. Hypoxic HNSCC-derived exosomal miR-5100 promoted the activation of CAFs by orchestrating QKI/AKT/STAT3 axis, which further facilitated HNSCC metastasis. Additionally, miR-5100 derived from plasma exosomes indicated HNSCC malignant progression. In conclusion, our findings illuminate a novel HIF1α/miR-5100/QKI pathway in HNSCC metastasis, and suggest that miR-5100 might be a potential biomarker and therapeutic target for HNSCC.

## Introduction

Head and neck cancer (HNC) is one of the most common human cancers worldwide, with a considerably high morbidity and mortality [1]. Head and neck squamous cell carcinoma (HNSCC) accounts for >90% of HNC, whose 5-year overall survival rate is only ~50% [2]. Local malignant invasion and lymph node (LN) metastasis, attributed to the interaction between tumor cells and tumor microenvironment (TME) [3, 4], are the major causes of poor prognosis of patients suffering from HNSCC. Therefore, in-depth exploration of the mechanisms of tumor-TME crosstalk-mediated metastasis is crucial for improving the outcome of HNSCC patients.

Hypoxia microenvironment ubiquitously exists in a variety of solid tumors, including HNSCC, because of the rapid tumor growth and inefficient blood supply, leading to the increasing stabilization of hypoxia-inducible factor 1α (HIF1α). Numerous studies have identified that HIF1α is implicated in tumor metabolism, angiogenesis, drug resistance, immune escape, and especially invasion-metastasis cascade by activating transcription of both coding RNAs as well as non-coding RNAs. In pancreatic cancer, hypoxia/HIF1α upregulates lncRNA BX111 to promote the transcription of ZEB1, a key regulator for epithelia–mesenchymal transition (EMT), to facilitate tumor metastasis and progression [5]. Besides, HIF1α-induced miR-1275 strengthens stemness and metastasis in lung adenocarcinoma by targeting multiple antagonists of Wnt/β-catenin and Notch pathways [6]. Given its importance, HIF1α is regarded as a master oncogene in tumor progression, and thus its regulatory mechanisms merit further investigation.

Accumulating evidence has shown that the communication between tumor cells and surrounding stromal cells in TME is also implicated in regulating cancer progression and therapy response [7, 8]. As the dominant cell type within the reactive stroma of many tumor types, cancer-associated fibroblasts (CAFs) are transformed from normal fibroblasts (NFs) by cancer cells through multiple mechanisms. Notably, microRNA reprogramming is one of the major contributors to CAFs transformation. It has been reported that the anti-miR-31, anti-miR-214, and pre-miR-155 promote the conversion of NFs into CAFs [9]. Activated CAFs further play a pro-oncogenic role mainly via secreting various cytokines, including TGF-β1, IL-6, and IL-8 [10, 11]. With the unraveling of the relationship between CAFs and tumor cells, CAFs are now being recognized as potential targets for anti-cancer therapy [12]. In this study, we found that both HIF1α and α-SMA (CAFs-specific marker) were expressed at high levels in HNSCC tissues with lymph node metastasis, but the potential role of tumor-CAFs crosstalk under hypoxia in HNSCC metastasis is still unclear.

Exosomes, secreted by multiple cell types, are a subtype of extracellular vehicles (EVs) that display a cup-shaped structure with a diameter of 40–160 nm approximately [13]. As important molecular vehicles, exosomes activate or repress various signaling pathways in the recipient cells by transmitting heterogeneous cargoes from donor cells to regulate numerous physiological and pathological processes, including cancer development. Recent studies have verified that tumor-derived exosomes (TDEs) play critical roles in TME remodeling, angiogenesis, drug resistance, invasion, and metastasis by delivering a wide range of functional proteins, mRNAs, and miRNAs [14]. Additionally, hypoxia could boost the release of exosomes by cancer cells and alter the miRNA profiles in TDEs to facilitate tumor progression. Exosomal miR-301a secreted by hypoxic pancreatic cancer cells mediates M2 macrophage polarization via the activation of PTEN/PI3Kγ pathway to accelerate tumor metastasis [15], and hypoxic tumor-derived exo-miR-135a-5p initiates favorable pre-metastatic niche formation to promote liver metastasis of colorectal cancer [16]. However, the molecular mechanisms that are responsible for HNSCC metastasis mediated by hypoxic TDEs remain poorly understood.

In this study, we illustrated that HIF1α overexpression correlated positively with LN metastasis and prognosis of HNSCC patients, indicating the critical role of hypoxia microenvironment in HNSC progression. Moreover, HIF1α strengthened the capacities of invasion and metastasis of HNSCC cells by upregulating miR-5100. Besides, QKI, an RNA-binding protein, was observably downregulated in CAFs compared with NFs and was closely linked with the suppression of CAFs activation. Exosomal miR-5100 secreted from hypoxic HNSCC cells plays a dominant role in regulating QKI expression in fibroblasts. Activated fibroblasts, in turn, further promoted the invasion-metastasis cascade in HNSCC. These results identified a novel mechanism by which HNSCC-TME crosstalk accelerated tumor metastasis and might provide new ideas to overcome metastasis in HNSCC.

## Materials and methods

### Clinical specimens

161 cases of HNSCC tissue specimens and 54 cases of preoperative plasma samples were obtained from patients who underwent surgery at Tianjin Medical University Cancer Hospital. All experiments performed on clinical samples were approved by the ethical committee of Tianjin Medical University Cancer Institute and Hospital (Approval number: Ek2020145).

### Cell culture and hypoxia treatment

HNSCC cell lines SCC15 and SCC25 were purchased from the American Type Culture Collection (ATCC, Manassas, VA, USA), and the TSCCA cell line was obtained from the Institute of Basic Medical Sciences, Chinese Academy of Medical Sciences. The HN4, HN6, and HN30 cell lines were generous gifts from Professor Wei Cao (Shanghai Jiaotong University, Shanghai, China), and the UM1 cell line was a gift from Professor Jinsong Hou (Sun Yat-sen University, Guangzhou, China). All of these cell lines were authenticated by short tandem repeat (STR) genotyping. The cell lines SCC15 and HN4 were maintained in DMEM/Ham’s F12 or DMEM, respectively, supplemented with 10% FBS in a humidified atmosphere (37 °C, 5% CO_2_). *Mycoplasma* contamination was detected in all cells before experiments. For hypoxia treatment, cells were maintained in a modulator incubator (Thermo Electron Co., Forma, MA, USA) in an atmosphere consisting of 94% N_2_, 5% CO_2_, and 1% O_2_.

### Antibodies and reagents

The following antibodies are used for immunohistochemistry (IHC), immunoblotting (IB), immunofluorescence (IF) and Chromatin immunoprecipitation (ChIP) assay in this study: HIF1α, Abcam (Cambridge, UK), ab51608 (IB, 1:1000; IHC, 1:400); α-SMA, Abcam, ab7817 (IB, 1:1000; IHC, 1:400; IF, 1:200); E-Cadherin, Proteintech (Rosemont, IL, USA), 60335-1-1 g (IB, 1:1000; IHC, 1:150); N-Cadherin, Proteintech, 22018-1-AP (IB, 1:1000; IHC, 1:200); Vimentin, Cell Signaling Technology (Danvers, MA, USA), #5641 (IB, 1:1000); Zeb1, Proteintech, 21544-1-AP (IB, 1:1000); Snail, Cell Signaling Technology, #3879 (IB, 1:1000); Slug, Cell Signaling Technology, #9585 (IB, 1:1000); Twist, Abcam, ab50581 (IB, 1:1000); QKI, Proteintech, 13169-1-AP (IB,1:1000; IHC, 1:400); ALIX, Santa Cruz (Dallas, Texas, USA), sc-53540 (IB, 1:1000); CD63, Santa Cruz, sc-5275 (IB, 1:1000); CD9, Santa Cruz, sc-13118 (IB, 1:500); AKT, Cell Signaling Technology, #4685 (IB, 1:1000); Phospho-AKT (Ser473), Cell Signaling Technology, #4060 (IB, 1:1000; IHC, 1:300); STAT3, Cell Signaling Technology, #9139 (IB, 1:1000); Phospho-STAT3 (Tyr705), Cell Signaling Technology, #9145 (IB, 1:1000; IHC, 1:300); Cortactin, Abcam, ab33333 (IF, 1:400); GAPDH, Santa Cruz, sc-365062 (IB, 1:5000) and Normal Rabbit IgG, Cell Signaling Technology, #2729 (ChIP). The Akt inhibitor MK2206 and STAT3 inhibitor Stattic were both purchased from Selleck (Shanghai, China).

### Transfection and transduction

MiR-5100 mimic and inhibitor, small interfering RNAs (siRNAs) targeting QKI, and negative control were purchased from RIBOBIO (Guangzhou, China). Lentivirus for miR-5100 knockdown and the control were obtained from Genechem (Shanghai, China). The full length of HIF1α was cloned into pLVX-IRES-neo vector. The shRNA sequence targeting HIF1α was 5′-CCGCTGGAGACACAATCATAT-3′, which was cloned into the pSIH1-H1-puro vector. Transfection, lentivirus package, and transduction were performed as previously described [17].

### RNA isolation and quantitative real-time PCR

Total RNA was extracted from cells and exosomes by using TRIzol Reagent (Invitrogen, Waltham, MA, USA) according to standard instructions. PrimeScript™ RT Master Mix (TaKaRa, Shiga, Japan) and GoScript™ Reverse Transcription System (Promega, Madison, WI, USA) were used to transcribe RNA to cDNA reversely. MiR-5100 was reversely transcribed using gene gene-specific primer, while U6 and mRNAs were converted using random hexamers. Quantitative Real-time PCR (qPCR) was performed using SYBR Premix Ex Taq^TM^ II (TaKaRa) on 7500 Real-Time PCR system (Applied Biosystems, Foster City, CA, USA). U6 and GAPDH were used as loading controls for miRNAs and mRNAs, respectively. Data was then normalized to the expression of control, and 2^−ΔΔCt^ method was used to determine the relative abundance of genes. The primers for reverse transcription and qPCR were listed in Supplementary Table S1.

### Small RNA sequencing

Two biologically independent RNA samples derived from HNSCC cells with hypoxia treatment for 16 h were extracted using TRIzol reagent. Small RNA sequencing was performed by Mingma Technologies (Shanghai, China) using Illumina HiSeq X10. After another RNA filtering, clean reads were mapped to the human genome using miRBase (v21). Then, the differential expression of known miRNAs was analyzed by edgeR. FC (Fold Change) > 2 and FDR (False Discovery Rates) < 0.05 were included into the criteria to screen out the significantly expressed miRNAs.

### ChIP

ChIP assay was performed by using EZ-Magna ChIP™ A/G ChIP Kit (Merck Millipore, Billerica, MA, USA) according to the manufacturer’s instructions. Interactions of HIF1α with the promoter of miR-5100 were measured by PCR amplification. The ChIP-PCR primers are shown in Supplementary Table S1.

### Isolation of exosomes

Exosomes were isolated from the conditioned medium using an ultracentrifugation method at 4 °C. The supernatant was centrifugated at 300 × *g* and 3000 × *g* to remove cells and cell debris, respectively. Then, the shedding vesicles and the other larger vesicles were removed with two runs of centrifugation at 10,000 × *g* for 80 min. Finally, the collected exosomes from the pellet were resuspended in PBS. The exosomes from the plasma of HNSCC patients before treatment were isolated by using ExoQuick Exosome Precipitation Solution (SBI System Biosciences) according to the manufacturer’s instructions.

### Transmission electron microscopy (TEM)

After Fixed with glutaraldehyde, 5–10 μl of exosomal solution were dropped to the copper mesh at room temperature for 10 min, and then filter paper was used to absorb excess liquid. 10 μl 2% phosphotungstic acid solution was added to the copper mesh for 2 min at room temperature. The transmission electron microscope HT7700 (HITACHI, Tokyo, Japan) was used to observe the exosomes and save digital images.

### Nanoparticle tracking analysis (NTA)

The size and density of exosomes were directly tracked using the Nanosight NS300 system (NanoSight technology, Malvern, UK) equipped with a 488 nm laser. Exosomes were resuspended in PBS, and then manually loaded into the sample chamber at ambient temperature. Each sample was measured in triplicate using a high-sensitivity scientific CMOS camera (sCMOS).

### Isolation of CAFs and NFs

CAFs and NFs were isolated from HNSCC tumor tissues and paired with adjacent normal tissues, respectively. In brief, the fresh tissues collected after surgery were immediately treated with PBS containing 20% penicillin/streptomycin for 2 h. Then, the tissues were minced to isolate fibroblasts which got attached to the culture dish and were further cultivated in DMEM supplemented with 10% FBS. The NFs/CAFs were identified by a CAFs-specific marker, α-SMA.

### Exosome labeling and tracking

Purified exosomes isolated from the conditioned medium were labeled with PKH26 Red Fluorescent Cell Linker Mini kit (Sigma-Aldrich, St. Louis, MO, USA) according to manufacturer’s instructions. Then, the labeled exosomes were added to NFs for exosome uptake. After incubation for 12 h, cells were observed by fluorescence microscopy.

### Immunofluorescence

Cells were plated on 18-mm cover glasses overnight for adherence. Then, the cells were fixed with 4% paraformaldehyde for 30 min, permeabilized with 0.2% Triton X-100 for 10 min, and blocked with 2% BSA for 1 h. Cells were incubated with primary antibodies overnight at 4 °C. The next day, cells were incubated with a secondary antibody conjugated to Alexa Fluor 488 or Alexa Fluor 594 (Cell Signaling Technology) for 1 h at room temperature. F-actin was visualized by TRITC Phalloidin (Yeasen, Shanghai, China), and the nuclei were stained with DAPI (Thermo Fisher Scientific, Waltham, MA, USA). All images were obtained using LSM 880 laser scanning confocal microscope (Zeiss, Oberkochen, Germany).

### Immunoblotting

Cells were lysed in RIPA buffer supplemented with protease and phosphatase inhibitors (APExBIO, Houston, Texas, USA). After quantification and denaturation, the proteins were separated in SDS-PAGE gels and transferred onto PVDF membranes (Merck Millipore). The membranes were blocked in 5% non-fat milk for 1 h at room temperature and then incubated with primary antibodies overnight at 4 °C. After incubation with secondary antibodies for 1 h, the membranes were visualized with an enhanced chemiluminescence kit (Cell Signaling Technology) on ImageQuant LAS4000 System (GE, Fairfeld, Connecticut, USA).

### Transwell assay

For invasion or migration assay in vitro, SCC15 or HN4 cells were planted into transwell inserts (3422, 8 μm, Corning, NY, USA) pre-coated with Matrigel (BD, Franklin Lakes, NJ, USA) or not. The bottom chambers were filled with 600 μl medium supplemented with 20% FBS. After incubation for 24 h, the penetrated cells were fixed with methanol and stained with 0.1% crystal violet. At least three fields were randomly obtained using an inverted microscope for cell counting.

### Wound healing assay

HNSCC cells were seeded into six-well plates. Until the cells grew nearly to 100% confluence, the scratches were made by using 10 μl pipette tips. Then, the cells were cultured in a fresh medium without FBS for another 24 h. At the indicated time point for 0 and 24 h, each image for the gaps was collected using an inverted microscope.

### Enzyme-linked immunosorbent assay (ELISA)

The conditioned medium of fibroblasts was collected after incubation with exosomes for 48 h. The concentration of IL-6, IL-8, and TGF-β was detected by a human ELISA kit (DAKEWE, Beijing, China) according to the manufacturer’s directions.

### Luciferase reporter assay

The promoter of miR-5100 was cloned into the upstream of firefly luciferase cassette in pGL3-Basic, and the wildtype/mutant binding site of miR-5100 in 3′-UTR of QKI was cloned into the downstream of firefly luciferase cassette in pIS0. The pGL3-Basic plasmid containing miR-5100 promoter was co-transfected with a Renilla luciferase vector (pRL-TK) into SCC15 cells under hypoxia treatment, while the pIS0 plasmid containing wildtype or mutated miR­5100 recognized sites was co­transfected with pRL-TK and miR-5100 mimic or negative control oligos into SCC15 or CAF1 cells, respectively. After 24 h, luciferase activities were measured by using Dual­Luciferase Assay kit (Promega) from three parallel wells according to standard instructions.

### IHC and ISH

For IHC staining, paraffin-embedded HNSCC tissues were routinely deparaffinized and then incubated with 0.3% H_2_O_2_ at room temperature for 30 mins. After antigen retrieval and non-specific antigen blocking, the sections were incubated with primary antibodies overnight at 4 °C. Next, the sections were visualized using Vectastain ABC kit and DAB chromagen (ZSGB-BIO, Beijing, China). For ISH assay, the specimens were incubated with miRNAscope^TM^ Probe-SR-Hs-pre-MIR-5100-S1, and stained using miRNAscope^TM^ HD detection reagents-RED (Advanced Cell Diagnostics, Newark, CA) according to the manufacturer’s instructions.

### Co-culture

HNSCC cells were planted into transwell inserts (3412, 0.4 μm, Corning) and co-cultured with pre-treated NFs or CAFs seeded in the bottom chambers for 48 h. Then, the HNSCC cells were collected for functional experiments or IB detection.

### Animal experiments

All animal protocols were approved by the Animal Care and Use Committee of Tianjin Medical University Cancer Institute and Hospital (Approval number: NSFC-AE-2020194). The HNSCC cells expressing control, HIF1α without/with miR-5100 shRNA were labeled as NC, HIF1α, and HIF1α+shmiR-5100, respectively. To evaluate the function of HIF1α/miR-5100 in vivo, 5 × 10^5^ indicated SCC15 cells were injected into the instep of 5-week-old BALB/c nude mice (7 mice per group) double-blindly and randomly. Tumor volume and body weight were measured every 3 days. The tumor volume was calculated using a formula (volume = (long diameter × short diameter^2^)/2). For detecting the effect of CAFs on HNSCC metastasis, a mass of mixed cells containing 4.5 × 10^5^ SCC15 cells and 1.5 × 10^5^ CAFs expressing control or shmiR-5100 (labeled as shNC and shmiR-5100) were injected into the instep of BALB/c nude mice (5 weeks old, 7 mice per group) double-blindly and randomly. Until 6-8 weeks, the xenograft tumors, popliteal lymph nodes, and inguinal lymph nodes were collected for H&E and IHC staining.

### Statistical analysis

All experiments other than IHC and animal experiments were repeated three times at least. Data were presented as mean ± standard deviation and then analyzed by using unpaired or paired Student’s *t* test. The statistical analyses were performed using GraphPad Prism 6, and differences with *P* < 0.05 were considered statistically significant.

## Results

### HIF1α is implicated in HNSCC metastasis, and its overexpression predicts poor prognosis

**Fig. 1:**
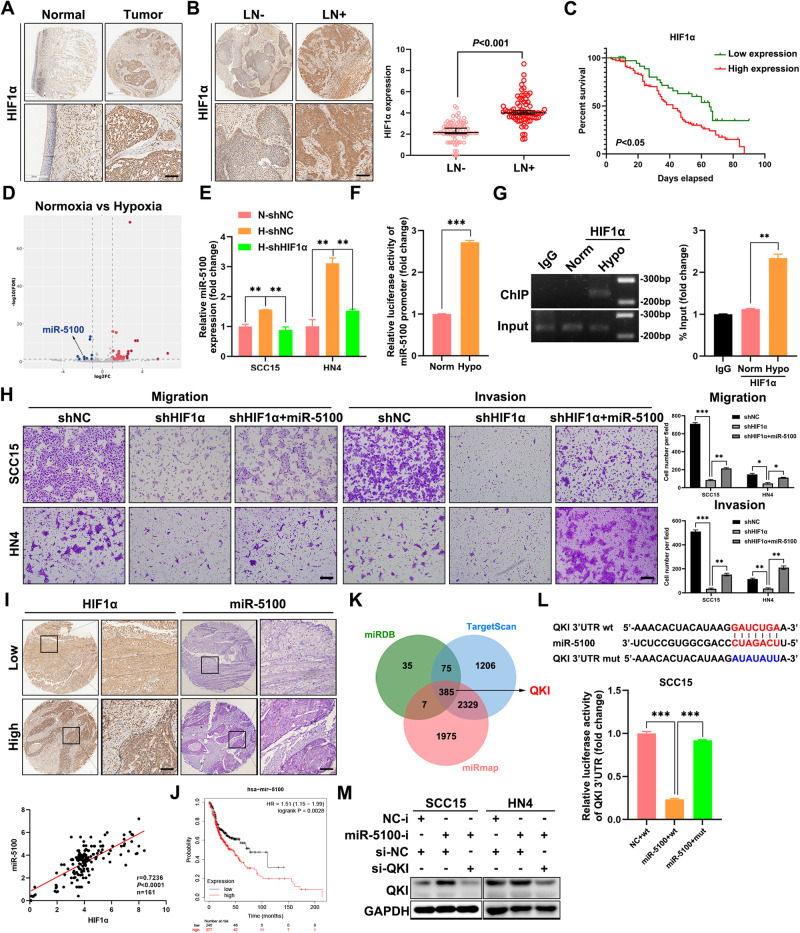
The HIF1α/miR-5100/QKI axis is implicated in the migration and invasion of HNSCC cells in vitro. **A** Representative IHC images of HIF1α in HNSCC specimens and adjacent normal tissues. Scale bar, 100 μm. **B** Representative IHC images showed that the expression of HIF1α in HNSCC specimens with lymph node metastasis was significantly higher than that in the ones without lymph node metastasis. LN-, without lymph node metastasis. LN+, with lymph node metastasis. Scale bar, 100 μm. **C** Kaplan–Meier survival curve of 92 HNSCC patients suggested that high expression of HIF1α indicated poor prognosis in HNSCC. **D** The volcano plot revealed differentially expressed microRNAs between normoxic SCC15 and hypoxic SCC15 cells. **E** The level of miR-5100 was detected in the SCC15 and HN4 cells expressing shNC or shHIF1α under normoxia/hypoxia using qPCR assay. N-shNC, HNSCC cells expressing shNC under normoxia. H-shNC, HNSCC cells expressing shNC under hypoxia. H-shHIF1α, HNSCC cells expressing shHIF1α under hypoxia. **F** Luciferase reporter assay was performed to assess the effect of hypoxia on the activity of miR-5100 promoter. Bar, SD for triplicate wells. **G** The result of ChIP assay indicated that hypoxia dramatically increased the binding of HIF1α to miR-5100 promoter. **H** The effect of miR-5100 on migration and invasion abilities of HNSCC cells expressing HIF1α shRNA by using transwell assay. The penetrated cells were stained and counted, and representative images were shown. Scale bar, 100 μm. **I** Representative IHC and ISH images identified a positive correlation between HIF1α and miR-5100 expression in HNSCC tissues. Scale bar, 100 μm. **J** The correlation between the expression of miR-5100 and overall survival in HNSCC was assessed via Kaplan–Meier plotter database. HR, hazard rate. **K** The putative targets of miR-5100 were predicted using three online algorithms (miRDB, miRmap, and TargetScan). **L** The predicted binding site of miR-5100 in the 3′UTR of QKI was cloned into the downstream of luciferase and the mutation was constructed as well. Dual-luciferase reporter assay showed that miR-5100 bound directly to the 3′-UTR of QKI in SCC15 cells. **M** The abundance of QKI was measured by immunoblotting in indicated groups. NC-i, negative control for microRNA inhibitor. miR-5100-i, miR-5100 inhibitor. Data in this figure, mean ± SD, *P < 0.05, **P < 0.01, ***P < 0.001. Norm, normoxia. Hypo, hypoxia.

To identify the clinical significance of HIF1α in HNSCC, IHC staining was performed to detect the expression of HIF1α in 161 HNSCC tissues. As shown in Fig. 1A, HIF1α expression was dramatically higher in tumor tissues than in adjacent normal tissues, which was consistent with the results of the analyses of TCGA and GEO HNSCC databases (Supplementary Fig. 1A, B). Importantly, HIF1α expression positively correlated with lymph node metastasis in HNSCC (Fig. 1B), indicating a potential role of HIF1α in the invasion-metastasis cascade of HNSCC. The Kaplan–Meier analysis suggested that HNSCC patients with higher HIF1α levels showed a worse outcome (Fig. 1C). To clarify the role of HIF1α in HNSCC metastasis, loss-of-function assay was then conducted in SCC15 and HN4 cells expressing shNC or shHIF1α (Supplementary Fig. 2A, B). Once HIF1α was depleted, the abilities of migration and invasion of HNSCC cells were notably weakened (Supplementary Fig. 2C). Additionally, compared with the negative control, the healing velocity of HIF1α-silenced HNSCC cells was retarded within 24 h (Supplementary Fig. 2D). Besides, HIF1α knockdown improved the expression of E-cadherin but suppressed the mesenchymal markers including N-cadherin, Vimentin, Zeb1, Snail and Slug (Supplementary Fig. 2E), which confirmed that HIF1α promoted EMT. Taken together, these results demonstrate that HIF1α acts as a crucial contributor to metastasis and unfavorable prognosis in HNSCC.

### Hypoxia activates miR-5100 transcription in a HIF1α-dependent manner to improve invasion of HNSCC cells in vitro

To explore the specific mechanism by which HIF1α promotes HNSCC metastasis, small RNA sequencing was performed using SCC15 cells under normoxia or hypoxia. The volcano plot showed that 9 microRNAs were significantly upregulated in hypoxia group compared with normoxia group (Fig. 1D). Among these candidates, miR-5100 was increased when HNSCC cells were exposed to hypoxia, while HIF1α knockdown reduced the level of miR-5100 in hypoxic SCC15 and HN4 cells (Fig. 1E), revealing a positive correlation between hypoxia/HIF1α and miR-5100. Subsequently, a remarkable increase of miR-5100 promoter activity was observed in HNSCC cells under hypoxia (Fig. 1F). In the meanwhile, hypoxia dramatically improved the binding of HIF1α to miR-5100 promoter (Fig. 1G). These results suggested that hypoxia upregulated HIF1α to activate the transcription of miR-5100.

Next, the effect of miR-5100 on the motility of HNSCC cells was measured. Upon delivery of miR-5100 mimic into SCC15 and HN4 cells (Supplementary Fig. 3A), the capacities of migration and invasion of these recipient cells were significantly reinforced relative to those control cells (Supplementary Fig. 3B, C). Enhanced miR-5100 notably improved the co-localization between F-actin and Cortactin along the cellular membrane (Supplementary Fig. 3D), which promotes the formation of invadopodia. Furthermore, miR-5100 mitigated the shHIF1α-mediated inhibition on migration, invasion, and EMT of HNSCC cells (Supplementary Fig. 4A–C and Fig. 1H). In addition, the expression of miR-5100 correlated positively with HIF1α level in HNSCC tissues according to the results of IHC and ISH assays (Fig. 1I), and the analysis of Kaplan-Meier Plotter database showed that higher miR-5100 expression predicted shorter overall survival of HNSCC patients (Fig. 1J). Collectively, these data suggest that hypoxia/HIF1α-induced miR-5100 strengthens the invasion of HNSCC cells in vitro.

### QKI is the bona fide target of miR-5100

In order to elucidate the pro-metastatic mechanism of miR-5100, we analyzed in silico the downstream targets of miR-5100 using three online algorithms, including miRDB, TargetScan, and miRmap (Fig. 1K). Among 385 candidates, QKI, a vital regulator in RNA splicing and maturation [18], was selected as a potential target of miR-5100 for further observation. The results of luciferase reporter assay showed that miR-5100 significantly reduced the luciferase activity of reporter plasmid containing putative miR-5100 binding sites from QKI 3′ UTR in SCC15 cells; whereas mutation of the binding sites restored the luciferase activity (Fig. 1L). Additionally, silence of endogenous miR­5100 in SCC15 and HN4 cells could enhance the expression of QKI (Fig. 1M). Moreover, QKI knockdown revived the invasion, migration, and EMT in miR-5100-depleted HNSCC cells (Supplementary Fig. 5A–C). These results indicate that miR-5100 promotes HNSCC invasion by targeting QKI.

### HIF1α/miR-5100 axis contributes to the invasion-metastasis cascade of HNSCC cells

**Fig. 2:**
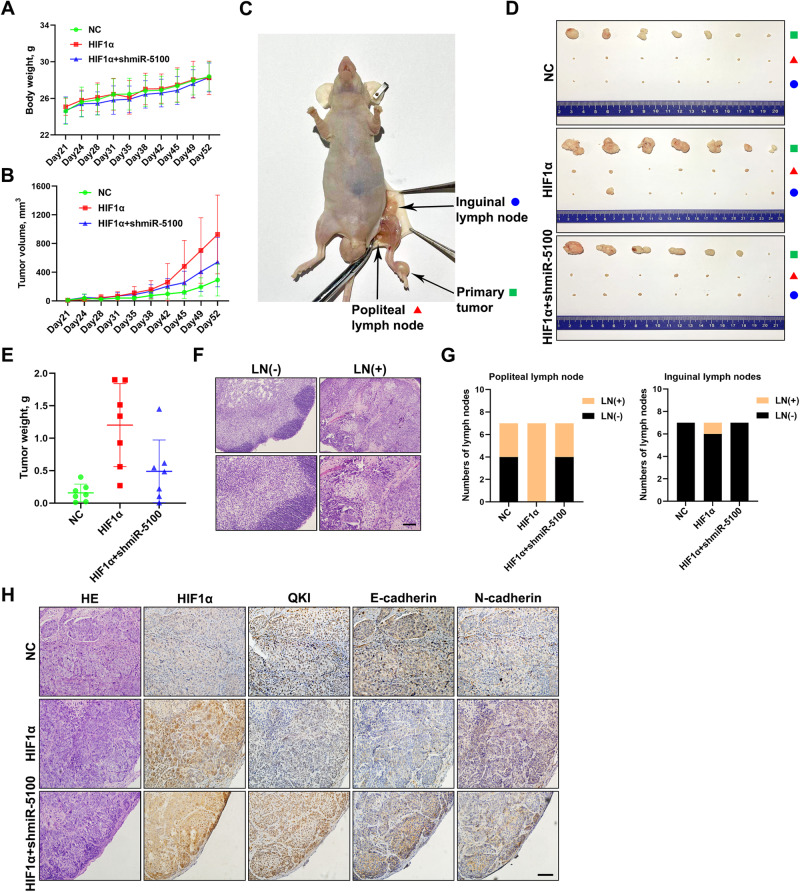
HIF1α-induced miR-5100 promotes LN metastasis of HNSCC cells. **A**, **B** Body weight and tumor volume were measured every 3 days to monitor the physical condition of mice and the growth rate of xenografts in indicated groups. **C**–**E** Representative images of primary tumors, popliteal and inguinal lymph nodes in indicated groups were presented, and the tumor weight was quantified (**E**). The depletion of miR-5100 dramatically restrained the HIF1α-induced increase in the size and weight of xenografts. **F** Representative images of hematoxylin-eosin staining of negative and positive lymph nodes. Scale bar, 100 μm. **G** The popliteal (left) and inguinal (right) lymph node metastases were recorded according to the results of HE staining. **H** IHC assay was performed in xenograft tissues to detect the levels of HIF1α, QKI, E-cadherin, and N-cadherin in indicated groups. Scale bar, 100 μm. Data in this figure, mean ± SD. LN (−), negative lymph node. LN (+), positive lymph node.

Then, the effect of HIF1α and miR-5100 on lymphatic metastasis in HNSCC was investigated in vivo using a lymph node metastasis mouse model. The SCC15 cells stably expressing control, HIF1α, or HIF1α plus shmiR-5100 were inoculated into the insteps of BALB/c nude mice. We found that the inhibition of miR-5100 attenuated HIF1α-mediated rapid growth of HNSCC xenografts, while the body weight of mice had no significant difference among these three groups (Fig. 2A, B). After 52 days, the tumor xenografts, popliteal and inguinal lymph nodes were collected (Fig. 2C). As shown in Fig. 2D, E, HIF1α overexpression resulted in a considerable increase in size and weight of xenografts, but the increase was dramatically restrained once miR-5100 was inhibited. Subsequently, HE staining was performed to assess lymph node metastasis (Fig. 2F), and we observed that miR-5100 knockdown hindered tumor metastasis in both popliteal and inguinal lymph nodes, which was obviously promoted by HIF1α (Fig. 2G). Furthermore, IHC staining showed that shmiR-5100 reversed HIF1α-induced regulation of the expression of QKI, E-cadherin, and N-cadherin in HNSCC xenografts (Fig. 2H), confirming the effect of HIF1α/miR-5100 axis on QKI and EMT process. Together, these findings suggest that HIF1α promotes lymphatic metastasis of HNSCC cells by regulating miR-5100.

### Hypoxic HNSCC-derived exosomes transform NFs into CAFs

**Fig. 3:**
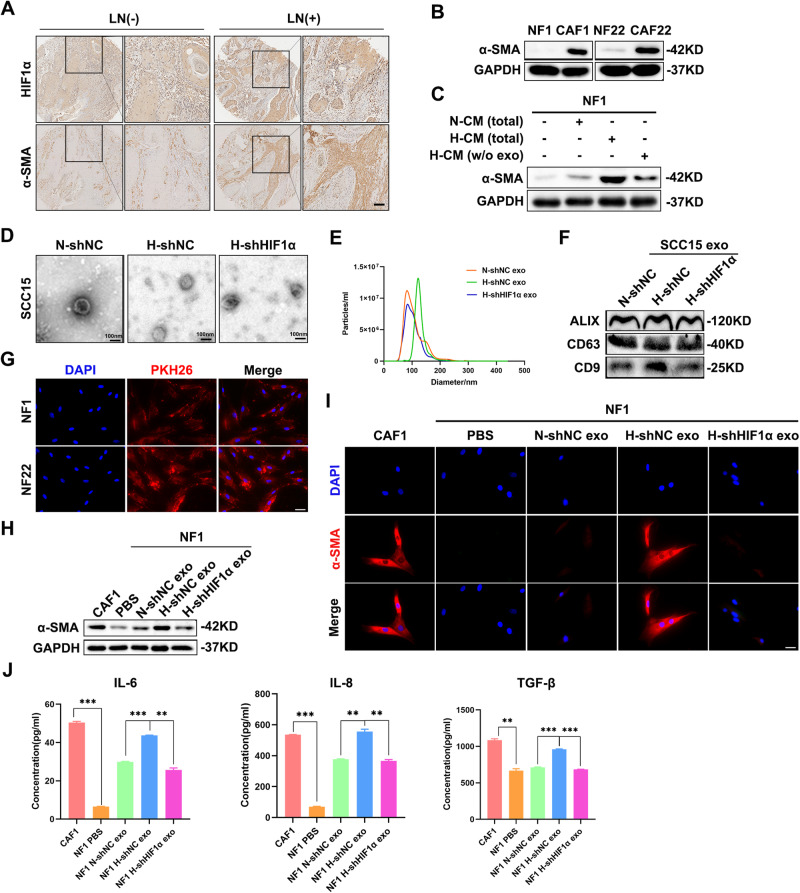
Hypoxic HNSCC-derived exosomes contribute to CAFs transformation. **A** The expression levels of HIF1α and α-SMA in HNSCC tissues with/without lymph node metastasis were measured using IHC staining. Scale bar, 100 μm. **B** Immunoblotting was conducted to determine the expression of α-SMA in paired primary NFs and CAFs isolated from HNSCC tissues and paired adjacent normal tissues. **C** The abundance of α-SMA was probed in NF1 cells treated with various conditioned medium as indicated. N-CM (total), normoxic SCC15 cells-derived conditioned medium. H-CM (total), hypoxic SCC15 cells-derived conditioned medium. H-CM (w/o exo), hypoxic SCC15 cells-derived conditioned medium without exosomes. **D**, **E** Transmission electron micrograph (**D**) and NanoSight analysis (**E**) of exosomes derived from SCC15 cells expressing shNC or shHIF1α under normoxia/hypoxia. Scale bar, 100 nm. **F** The protein expressions of exosomes markers ALIX, CD63, and CD9 were detected by immunoblotting assay in exosomes derived from SCC15 cells expressing shNC or shHIF1α under normoxia/hypoxia. **G** Internalization of exosomes by NF1 cells was examined by immunofluorescence. Scale bar, 20 μm. **H**, **I** The level of α-SMA was detected using immunoblotting and immunofluorescence in CAF1 and NF1 treated with PBS or exosomes as indicated. Scale bar, 20 μm. **J** The secretion of IL-6, IL-8, and TGF-β in CAF1 and indicated treated NF1 cells was measured using ELISA. Data, mean ± SD, **P < 0.01, ***P < 0.001.

As the predominant stromal cell type in TME, CAFs have been demonstrated to actively participate in cancer metastasis through interaction with tumor cells [19]. Similarly, we found that the staining of α-SMA, the most effective marker of CAFs, was obviously stronger in HNSCC specimens with lymph node (LN) metastasis than in the ones without LN metastasis (Fig. 3A). It is worth noting that there was a potential correlation between the level of α-SMA in CAFs and HIF1α expression in HNSCC cells (Fig. 3A), indicating that CAFs activation was likely attributed to HNSCC cells under hypoxia. In order to confirm the hypothesis, the phenotypic experiments were performed using primary CAFs and NFs extracted from HNSCC tissues and paired adjacent normal tissues (Fig. 3B). The IB results showed that compared with the conditioned medium (CM) of normoxic HNSCC cells, the hypoxic tumor cells-derived CM (hCM) markedly improved the expression of α-SMA in NFs; while the removal of exosomes from hCM severely limited the increase (Fig. 3C and Supplementary Fig. 6A), which implied that hypoxic HNSCC-derived exosomes might play a prominent role in converting NFs to CAFs. Then, the indicated exosomes, derived from CM of SCC15 cells expressing shNC or shHIF1α under normoxia/hypoxia (N-shNC, H-shNC, H-shHIF1α), were isolated and identified by TEM and NTA (Fig. 3D, E). Moreover, the exosomes were further verified through the detection of ALIX, CD63 as well as CD9 (Fig. 3F). The images of IF revealed that HNSCC cells-derived exosomes labeled by PKH26 could be internalized by the NFs successfully (Fig. 3G). As shown in Fig. 3H, I, compared to the exosomes secreted by normoxic SCC15 cells (N-shNC exo), hypoxic SCC15 cells-derived exosomes (H-shNC exo) notably upregulated the level of α-SMA in NF1 cells; by contrast, no significant increase of α-SMA expression appeared in NF1 cells exposed to the exosomes from HIF1α-depleted SCC15 cells under hypoxia (H-shHIF1α exo), which revealed the crucial effect of HIF1α on exosome function. Similar results could be observed in NF22 cells with the same treatment (Supplementary Fig. 6B, C). Additionally, just like CAFs, H-shNC exo-treated NFs released more pro-inflammatory cytokines, including IL-6, IL-8, and TGF-β (Fig. 3J), which act as key players in remodeling the TMe and promoting carcinoma development. Altogether, the above results identify that hypoxic HNSCC-derived exosomes contribute to the transformation of NFs to CAFs in a HIF1α-dependent manner.

### Hypoxic HNSCC-derived exosomal miR-5100 induces the activation of CAFs

**Fig. 4:**
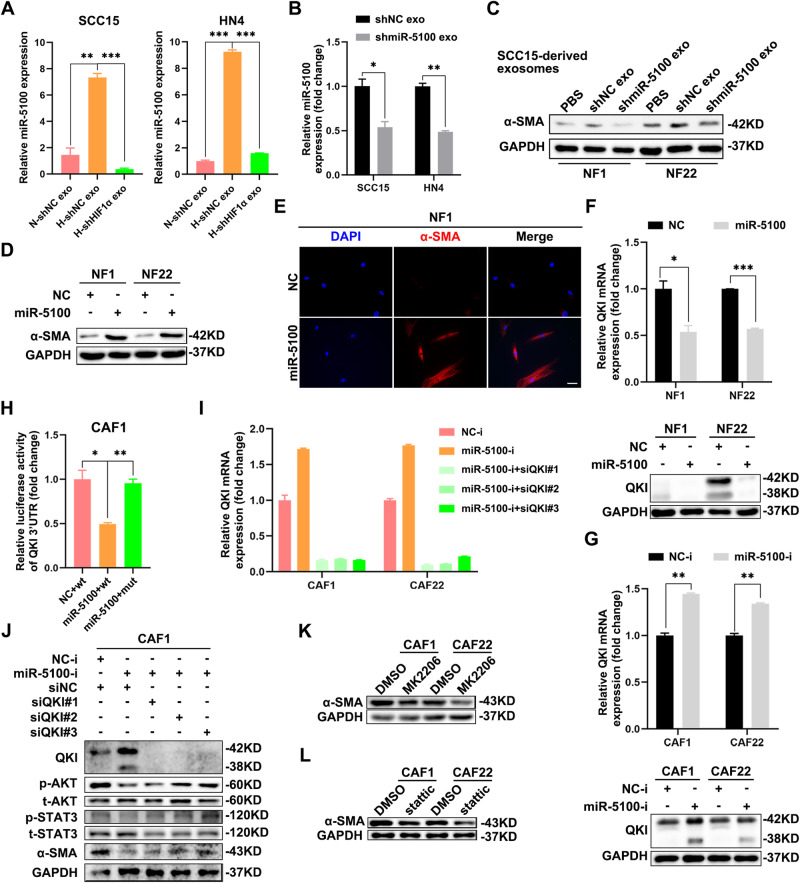
MiR-5100 in hypoxic HNSCC-derived exosomes mediates fibroblasts activation by modulating QKI/AKT/STAT3 pathway. **A** Relative expression of miR-5100 was detected using qPCR in exosomes from HNSCC cells expressing shNC or shHIF1α under normoxia/hypoxia. **B** The results of qPCR revealed that the expression of miR-5100 was reduced in exosomes from miR-5100-depleted SCC15 and HN4 cells. **C** NF1 and NF22 cells were treated with exosomes from miR-5100-silenced SCC15 cells, and then the abundance of α-SMA was measured using immunoblotting assay. **D**, **E** The expression of α-SMA in NF1 and NF22 cells transfected with miR-5100 mimic was examined through immunoblotting (**D**) and immunofluorescence (**E**). Scale bar in **E**, 20 μm. **F** The results of qPCR and immunoblotting showed that miR-5100 markedly inhibited QKI in NF1 and NF22 cells. **G** The mRNA and protein levels of QKI were enhanced when endogenous miR-5100 was knocked down in CAF1 and CAF22 cells. **H** Dual-luciferase reporter assay showed that miR-5100 bound directly to the 3′UTR of QKI in CAF1 cells. **I**, **J** The results of qPCR and IB showed that siQKI relieved the inhibitory effect of miR-5100 inhibitor on the expression of α-SMA and the activation of AKT and STAT3. **K**, **L** The expression of α-SMA was measured by using IB in CAF1 and CAF22 cells exposed to AKT inhibitor MK2206 (**K**) and STAT3 inhibitor Stattic (**L**). GAPDH was used as internal control. Data in this figure, mean ± SD, *P < 0.05, **P < 0.01, ***P < 0.001. NC-i, negative control for microRNA inhibitor. miR-5100-i, miR-5100 inhibitor.

In view of the critical role of miR-5100 in HNSCC progression, we further explored whether it could be transferred by exosomes to modulate the TME. As shown in Fig. 4A, hypoxia led to an elevated level of miR-5100 in the exosomes secreted by HNSCC cells, whereas HIF1α knockdown impaired the hypoxia-induced enhancement, indicating that both the cellular and exosomal miR-5100 expressions were regulated by hypoxia/HIF1α. Besides, miR-5100 depletion in HNSCC cells led to decreased levels of miR-5100 in the secreted exosomes (Supplementary Fig. 6D and Fig. 4B), which further verified the existence of miR-5100 in exosomes. As shown in Supplementary Fig. 6E, the higher level of miR-5100 in CAFs revealed the potential role of miR-5100 in CAFs activation. To determine whether exosomal miR-5100 activated fibroblasts, NFs were treated with indicated HNSCC-derived exosomes, respectively. Compared with the PBS group, TDEs (NC exo) facilitated the activation of fibroblasts as the CAFs associated marker, α-SMA was increased after incubation with the exosomes. Conversely, the tendency induced by NC exo was blocked on account of miR-5100 knockdown in HNSCC-derived exosomes (Fig. 4C and Supplementary Fig. 6F). Moreover, the level of α-SMA in NFs was significantly increased upon overexpression of miR-5100 (Supplementary Fig. 6G and Fig. 4D, E), whereas miR-5100 silencing produced the opposite results in CAF1 and CAF22 cells (Supplementary Fig. 6H–J). These results above proved that miR-5100 accounted for the effect of hypoxic HNSCC-derived exosomes on CAFs activation.

QKI is reported to be related to fibroblast activation in the human heart [20]. Therefore, we attempted to investigate whether QKI was also influenced by miR-5100 in fibroblasts and participated in CAFs activation. As shown in Supplementary Fig. 6K, L, the expression of QKI in CAFs was lower than that in NFs. Then, we found that delivery of miR-5100 mimic into NFs dramatically reduced the expression of QKI at both mRNA and protein levels (Fig. 4F). On the contrary, QKI was significantly upregulated in miR-5100-silenced CAFs (Fig. 4G). Additionally, the results of the dual-luciferase reporter assay further confirmed that miR-5100 could directly target QKI in CAF1 cells (Fig. 4H). As expected, siRNAs targeting QKI reversed the reduced level of α-SMA induced by miR-5100 inhibition in CAF1 cells (Fig. 4I, J). A recent study has identified that AKT and STAT3 pathways are indispensable for CAFs activation [21, 22]. Thus, we detected the activation of AKT and STAT3 in the recipient CAF1 cells. The silence of QKI significantly revived the phosphorylation of AKT and STAT3 abolished by miR-5100 knockdown (Fig. 4J). In the meanwhile, the expression level of α-SMA was decreased in CAFs exposed to AKT inhibitor MK2206 and STAT3 inhibitor stattic, respectively (Fig. 4K, L). Collectively, these data reveal that hypoxic HNSCC-derived exosomal miR-5100 promotes CAFs activation by regulating QKI/AKT/STAT3 signaling pathway.

### MiR-5100 activates fibroblasts to accelerate invasion and metastasis in HNSCC

**Fig. 5:**
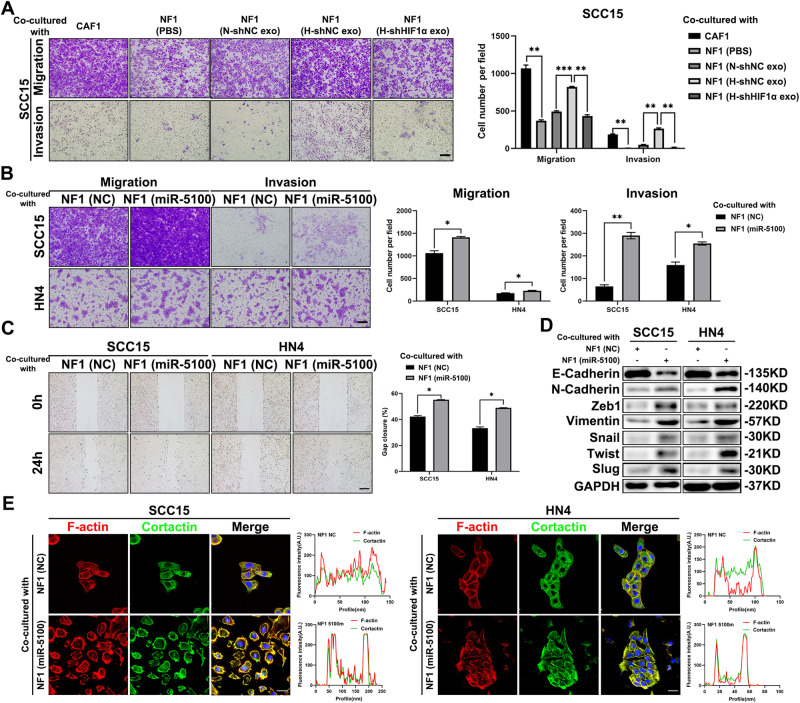
Activated fibroblasts strengthen motility capacity of HNSCC cells. **A** SCC15 cells were co-cultured with CAF1 and indicated treated NF1, and then the transwell assay was performed to assess the abilities of migration and invasion of SCC15 cells. Scale bar, 100 μm. **B**, **C** Transwell assay and wound-healing assay were conducted in SCC15 and HN4 cells after co-culture with miR-5100-elevated NF1 or negative control. Scale bar in **B**, 100 μm. Scale bar in **C** 200 μm. **D** The abundance of EMT-related markers was analyzed in indicated groups. **E** Immunofluorescence of F-actin and Cortactin in indicated groups. The Fluorescence intensities of F-actin and Cortactin along with the white lines, was measured. Scale bar, 20 μm. Data in this figure, mean ± SD, *P < 0.05, **P < 0.01, ***P < 0.001.

**Fig. 6:**
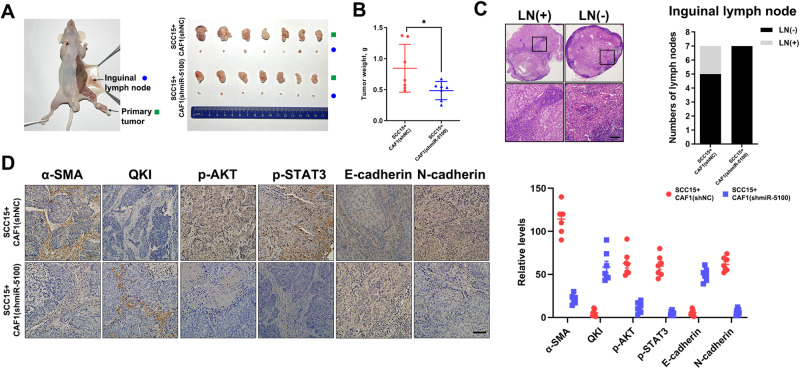
Inhibition of miR-5100 in CAFs decreases LN metastasis of HNSCC cells. **A** SCC15 cells mixed with CAF1 expressing shNC or shmiR-5100 were injected into the insteps of nude mice. Representative images of primary tumors and inguinal lymph nodes in these two groups were presented. **B** The tumor weight was quantified in the groups as described in **A**. **C** HE staining was used to determine the inguinal lymph node metastasis in indicated groups. LN (+), positive lymph node. LN (−), negative lymph node. **D** The levels of α-SMA, QKI, p-AKT, p-STAT3, E-cadherin, and N-cadherin in the xenograft tissues of indicated groups were measured by using IHC staining. Scale bar, 100 μm. Data in this figure, mean ± SD, *P < 0.05.

To investigate the impact of activated fibroblasts on HNSCC progression, we then performed co-culture assays in vitro using HNSCC cells with CAF1 or NF1 cells treated by TDEs. As shown in Fig. 5A, the capacities of migration and invasion were significantly strengthened in SCC15 cells co-cultured with CAF1 cells and H-shNC exo-educated NF1 cells compared to the other three groups. Similarly, miR-5100-overexpressed NF1 cells exhibited a greater ability to promote migration and invasion of HNSCC cells in vitro (Fig. 5B, C), and also promoted the EMT process as the downregulation of E-cadherin and upregulation of N-cadherin, Zeb1, Vimentin, Snail, Twist and Slug in HNSCC cells (Fig. 5D). Besides, the co-culture with NF1 activated by miR-5100 improved the formation of invadopodia in both SCC15 and HN4 cells (Fig. 5E). To further elucidate the function of miR-5100 exerting in fibroblasts to modulate in vivo metastasis of HNSCC cells, SCC15 cells and CAF1 cells expressing the control or shmiR-5100 were mixed and injected into the insteps of BALB/c nude mice. Then, the HNSCC xenografts and inguinal lymph nodes were harvested (Fig. 6A). Compared with the negative control, inhibition of miR-5100 in CAFs obviously hindered tumor growth and lymph node metastasis of HNSCC cells (Fig. 6B, C). The IHC results showed that when miR-5100 was depleted, the expression of α-SMA was dramatically reduced, while an obvious increase in QKI level was observed in CAFs. Moreover, the activities of AKT and STAT3 were repressed in both tumor cells and fibroblasts, and the EMT process in HNSCC was also reversed according to the staining of E-cadherin and N-cadherin (Fig. 6D). Overall, these results corroborate that miR-5100-activated CAFs play a critical role in promoting HNSCC metastasis.

### Plasma exosomal miR-5100 serves as a promising biomarker in HNSCC

**Fig. 7:**
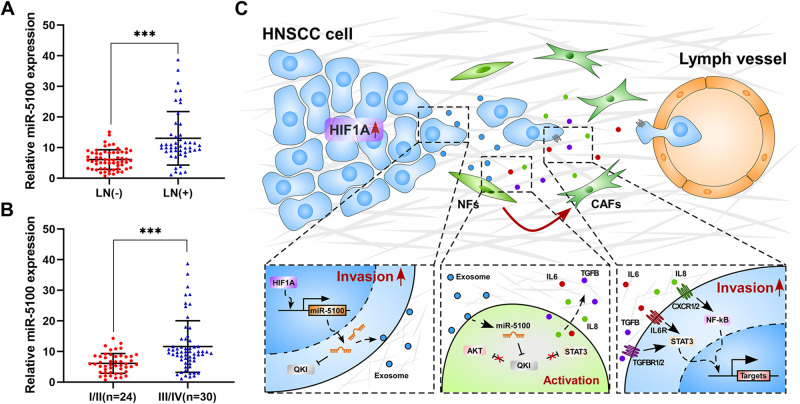
The level of plasma exosomal miR-5100 correlates with malignant progression of HNSCC. **A** Quantitative real-time PCR was performed to detect the expression of exosomal miR-5100 from plasma collected from HNSCC patients without LN metastasis (*n* = 30) and the ones with LN metastasis (*n* = 24). **B** The result of qPCR showed that compared with early-stage HNSCC (I/II stage, *n* = 24), the level of plasma exosomal miR-5100 was significantly increased in late-stage HNSCC (III/IV, *n* = 30). Data in this figure, mean ± SD, ***P < 0.001. **C** Schematic of the mechanism of hypoxia-induced miR-5100 for orchestrating tumor-CAFs crosstalk and HNSCC metastasis.

Increasing evidence demonstrates that exosomes enriched in body fluids have great promise to serve as novel biomarkers in liquid biopsy, a revolutionary strategy for cancer diagnosis and prognosis prediction characterized by minimally invasive detection [23]. Therefore, we further measured the level of miR-5100 in plasma exosomes obtained from HNSCC patients to evaluate its potential clinical application in HNSCC. The results revealed that the expression of plasma exo-miR-5100 was positively associated with lymph node metastasis (Fig. 7A, *P* < 0.001). Furthermore, patients with advanced HNSCC showed a significantly higher level of plasma exo-miR-5100 than those in early stages (Fig. 7B, I/II stages: 0.77–14.32 vs. III/IV stages: 1.16–38.71, *P* < 0.001), which indicated the effectiveness of plasma exo-miR-5100 for discriminating tumor stages of HNSCC patients. Taken together, these data identify a remarkable increase in plasma exo-miR-5100 level during HNSCC progression and suggest that exosomal miR-5100 may be utilized as a grading basis for HNSCC patients through liquid biopsy.

## Discussion

Hypoxia microenvironment has emerged as a crucial contributor to tumor progression in a HIF-dependent manner. Active HIF is a heterodimeric protein that consists of the constitutively expressed HIF1β subunit and an O2-dependent HIFα isoform (HIF1α, HIF2α, or HIF3α). Previous studies have identified HIF1α as the major isoform that indicates poor outcomes in HNSCC [24], which is consistent with the results of our research. In normoxia, HIF1α is regulated by the O2-dependent prolyl hydroxylase domain (PHD) proteins and the von Hippel-Lindau (VHL) protein, then rapidly degraded by the proteasome. As expected, we previously verified that VHL, as an E3 ligase, repressed both HIF1α and β-catenin to reverse EMT process in HNSCC [25]. The stability of HIF1α is greatly increased in hypoxia, and HIF induces transcription by binding to hypoxia-responsive elements (HREs) in the promoter of target genes, including microRNAs, to promote tumor malignancy. Jin et al. have illustrated that HIF1α-induced miR-23a∼27a∼24 cluster collectively regulates the glucose metabolic network in colorectal cancer [26]. Another research indicated that HIF1α-activated miR-17-5p promotes tumor growth and metastasis of gastric cancer by repressing PDCD4 [27]. In this study, we further explored the potential microRNAs regulated by HIF1α in HNSCC metastasis by using small RNA sequencing and clarified that miR-5100 served as a novel target of HIF1α. The results suggested that HIF1α could elevate miR-5100 expression by direct transcriptional activation, while MKL1 is the only known transcriptional factor of miR-5100 reported previously [28]. Two independent studies from lung cancer demonstrated that miR­5100 promoted growth and chemoresistance of neoplastic cells [29, 30], whereas miR­5100 overexpression decreased the aggressive phenotype of pancreatic cancer cells [31]. In HNSCC, the absence of miR-5100 impaired HIF1α-mediated growth and metastasis of malignant cells. These data support the doctrine that one specific microRNA may play various and even opposite roles in distinct genetic contexts and suggest that HIF1α-induced miR-5100 is crucial for HNSCC metastasis.

In addition to functioning intracellularly, microRNAs also could be secreted by donor cells to modulate neighboring and distant cells via exosomes. To a great extent, exosomes represent biological “snapshot” of parent cells according to the composition of their cargos. It has been reported that hepatocellular carcinoma-derived exosomal miR-23a-3p attenuates antitumor immunity by upregulating PD-L1 in macrophages [32]. Zeng and colleagues found that miR-25-3p could be transferred from colorectal cancer cells to endothelial cells to facilitate vascular permeability and angiogenesis, which finally enhances tumor metastasis [33]. Of note, hypoxia could promote the secretion of exosomes by cancer cells and regulate the manifest of cargos loaded into exosomes. As a consequence, exosomes released under hypoxia play a vital role in aiding tumor cells crosstalk with the microenvironment constituents to create conditions advantageous for cancer progression and metastatic spread. In multiple myeloma, miR-135b is upregulated in cancer-derived exosomes under hypoxia, and then transmitted to endothelial cells to enhance angiogenesis [34]. Another piece of evidence is the tumor-promoting role of exo-miR­1246 in glioma. Hypoxic glioma-derived exo-miR-1246 markedly induces M2 macrophage polarization, which subsequently promotes tumor proliferation, migration, and invasion [35]. In this report, we found that hypoxia/HIF1α induced the upregulation of both cellular and exosomal miR-5100 in HNSCC. Exosomal miR-5100, resembling the function of hepatocellular carcinoma-secreted exo-miR-1247-3p [11], was internalized by recipient fibroblasts to induce the transformation of CAFs, which further accelerated HNSCC progression. Our findings may partially explain the positive correlation between HIF1α expression in HNSCC cells and α-SMA level in CAFs, and enrich the underlying mechanisms of conversion from NFs to CAFs [36]. Meanwhile, these data provide a new perspective on the functions of hypoxia-activated miR-5100 in communication between HNSCC and TME, and suggest that targeting miR-5100 via various delivery ways, such as engineering exosomes may be a promising antitumor strategy.

The RNA-binding protein QKI, also known as Quaking, is a member of the signal transduction and activation of RNA (STAR) family, which also belongs to the heterogeneous nuclear ribonucleoprotein K (hnRNPK) homology domain protein family, a well-known regulator of pre-mRNA alternative splicing [37]. There are three major isoforms, including QKI-5, QKI-6, and QKI-7, which can regulate pre-mRNA splicing, transportation, or stability. A series of studies have further elucidated that QKI has key roles in cardiovascular development, cell differentiation, metabolism, and cancer progression. In lung cancer, QKI (QKI-5) suppresses cancer-associated aberrant splicing of NUMB to prevent the activation of Notch signal and tumor proliferation [38]. Besides, QKI could also regulate mRNA stability of target genes to repress tumor progression [39, 40]. However, recent reports from non-small cell lung cancer and esophageal squamous cell carcinoma indicated that QKI (QKI-5) functions as an oncogene to promote tumor malignancies [41, 42]. In this study, we confirmed that QKI, a target gene of miR-5100, suppressed the invasion and EMT process of HNSCC cells, which agrees with the results of the previous study [43]. Additionally, we first identified QKI downregulation in CAFs compared with NFs, and clarified the suppressive role of QKI in CAFs transformation by inhibiting AKT and STAT3 pathways, which are critical for CAFs conversion [21, 22]. Interestingly, Shi et al. illuminated that QKI inhibits the expression of HIF1α to suppress tumorigenesis of clear cell renal cell carcinoma [44]. Therefore, a potential HIF1α/miR-5100/QKI feedback loop may exist in HNSCC, which remains to be verified. The mechanisms of QKI regulating HNSCC progression and CAFs activation merit further exploration in the future.

Up to now, tissue biopsy which needs the extraction of small tumor tissues for histological analysis, has been identified as the most common method for cancer diagnosis. However, this invasive method is unfit to represent tumor heterogeneity or monitor tumor progression dynamically, and simultaneously increases the potential of metastasis. Liquid biopsy, as a minimally invasive even non-invasive strategy for cancer diagnosis through biofluids such as blood, saliva, and urine, has drawn widespread attention in recent years and has the advantage of real-time monitoring for cancer diagnosis, treatment planning, response evaluation, and prognosis prediction [45]. Currently, circulating tumor cells (CTCs), circulating tumor DNA (ctDNA) and exosomes have been considered as the three main biological components in liquid biopsy. Compared with CTCs and ctDNA, exosomes show superior advantages, including living-cell secreted vesicles, large amounts, and stable circulation [46]. A growing number of reports have indicated that exosomal miRNAs could suggest tumor origin and serve as promising biomarkers for multiple cancers. Zhou et al. demonstrated that exosomal miR-105 from the serum of patients with breast cancer could predict metastatic progression at an early stage before clinical detection of metastasis [47]. Plasma exo-miR-1260b is also reported to be a diagnostic and prognostic biomarker in non-small cell lung cancer [48]. Recent reports have shown that saliva could also be collected to detect exosomal miRNAs for diagnosis in HNSCC due to the close proximity of the tumor to saliva [49], which indicates that analyzing exo-microRNAs from both saliva and blood may be optimal for effective screening of HNSCC. Our findings in this study confirmed that exo-miR-5100 from plasma correlated positively with lymph node metastasis and clinical stages of HNSCC patients, suggesting that exosomal miR-5100 can be exploited as a valuable biomarker in HNSCC diagnosis. However, the detection of salivary exo-miR-5100 and the effect of exo-miR-5100 on prognosis prediction in HNSCC remain to be explored.

In summary, this study demonstrates that hypoxia microenvironment increases miR-5100 in HNSCC cells via HIF1α-mediated transcriptional activation. The cellular and exosomal miR-5100 could directly repress the expression of QKI in HNSCC cells and NFs respectively, leading to the reinforcement of invasion capacity of HNSCC cells and the conversion of CAFs which in turn further strengthens tumor malignancies partially through activating critical signaling in HNSCC (Fig. 7C). Therefore, the hypoxia-mediated tumor-CAFs crosstalk plays a crucial role in HNSCC invasion-metastasis cascade. Our data identify a novel HIF1α/miR-5100/QKI axis in HNSCC metastasis, and highlight the potential of miR-5100 as a promising target for diagnosis and treatment in HNSCC.

### Supplementary information

Supplementary materials

Full uncut gels

aj-checklist

## Data Availability

All data generated or analyzed during this study are included in this published article and supplementary information files.
