# Supplementary material for: Hypoxia-induced miR-5100 promotes exosome-mediated activation of cancer-associated fibroblasts and metastasis of head and neck squamous cell carcinoma
Source: Cell Death Dis. 2024 Mar 14;15(3):215. doi: 10.1038/s41419-024-06587-9 (PMC10940661; doi:10.1038/s41419-024-06587-9)
Supplement: Supplementary file 1 — Supplementary materials [file 41419_2024_6587_MOESM1_ESM.docx]

**Supplementary Table S1. List of primers sequence.**

| **Application** | **Sequence (5’🠪3’)** |
| --- | --- |
| **microRNA reverse transcription** |  |
| miR-5100 | CTCAACTGGTGTCGTGGAGTCGGCAATTCAGTTGAGAGAGGCAC |
| **qPCR detection** |  |
| HIF1α | F: GAACGTCGAAAAGAAAAGTCTCG |
|  | R: CCTTATCAAGATGCGAACTCACA |
| miR-5100 | F: ACACTCCAGCTGGGTTCAGATCCCAGCGGT |
|  | R: TGGTGTCGTGGAGTCG |
| QKI | F: CTGATGCTGTGGGACCTATTG |
|  | R: GTTGTTTGGCTGTAAGTCCTCT |
| GAPDH | F: TGCACCACCAACTGCTTAGC |
|  | R: GGCATGGACTGTGGTCATGAG |
| U6 | F: CTCGCTTCGGCAGCACA |
|  | R: AACGCTTCACGAATTTGCGT |
| **ChIP-PCR** |  |
| miR-5100 promoter containing | F: CATGGGTGGGGTGTCCCTGGT |
| HRE sequence | R: CCCCACCCCTCCTTGTGCATT |

**
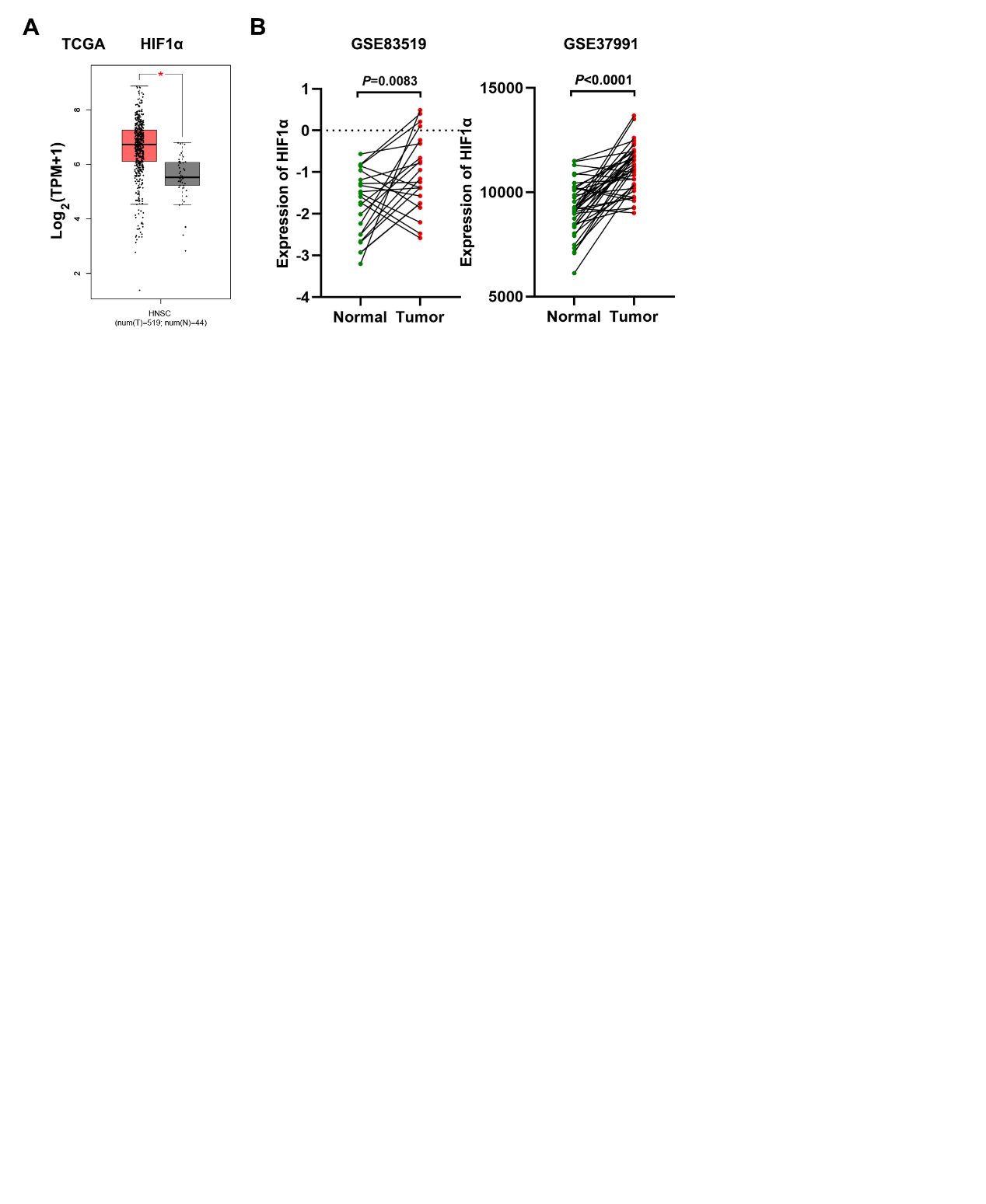
**

**Supplementary Fig. 1 HIF1α is overexpressed in HNSCC.** **A and B** The bioinformatic analysis of TCGA (**A**) and GEO (**B**, GSE83519 and GSE37991) databases showed that HIF1α was upregulated in HNSCC tissues compared with normal tissues. Data, mean ± SD, **P*<0.05. HNSC, head and neck squamous cell carcinoma. T, tumor tissues. N, normal tissues. TPM, transcripts per million.

**
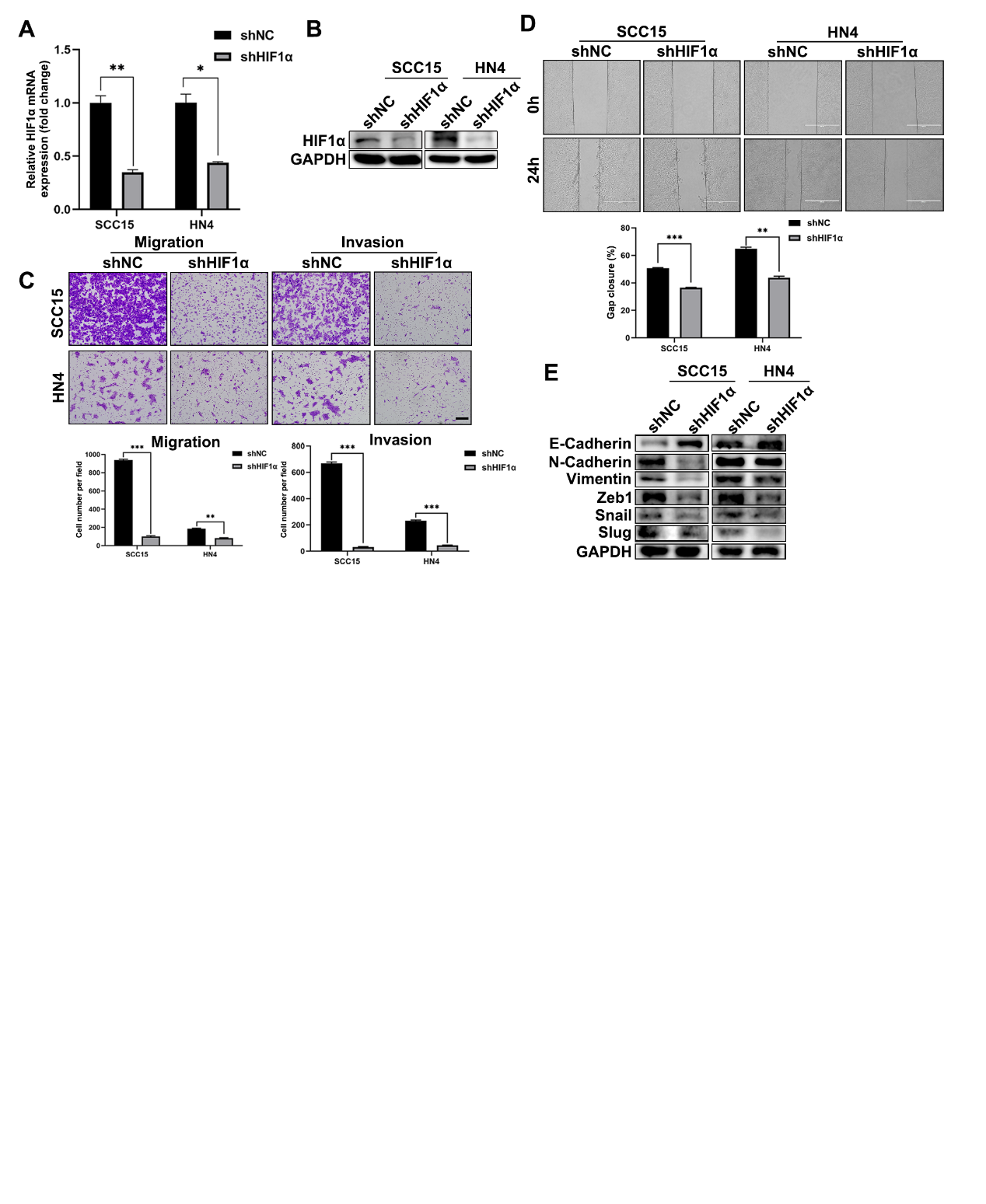
**

**Supplementary Fig. 2 Depletion of HIF1α impedes migration and invasion of HNSCC cells *in vitro*.** **A and B** The mRNA and protein expressions of HIF1α in both SCC15 and HN4 cells expressing HIF1α shRNA were measured using qPCR (**A**) and Immunoblotting assay (**B**). **C** Reduced expression of HIF1α made less SCC15 and HN4 cells relative to control counterparts to penetrate membrane in transwell assays. Scale bar, 100 μm. **D** Representative photographs of gap at the beginning (0 hour) and the endpoint (24 hour) of wound healing assay identified the inhibitory effect of shHIF1α on motility of SCC15 and HN4 cells. Scale bar, 400 μm. **E** The abundance of EMT-related markers was analyzed in SCC15 and HN4 cells expressing shNC and shHIF1α, respectively. Data in this figure, mean ± SD, **P*<0.05, ***P*<0.01, ****P*<0.001.

**
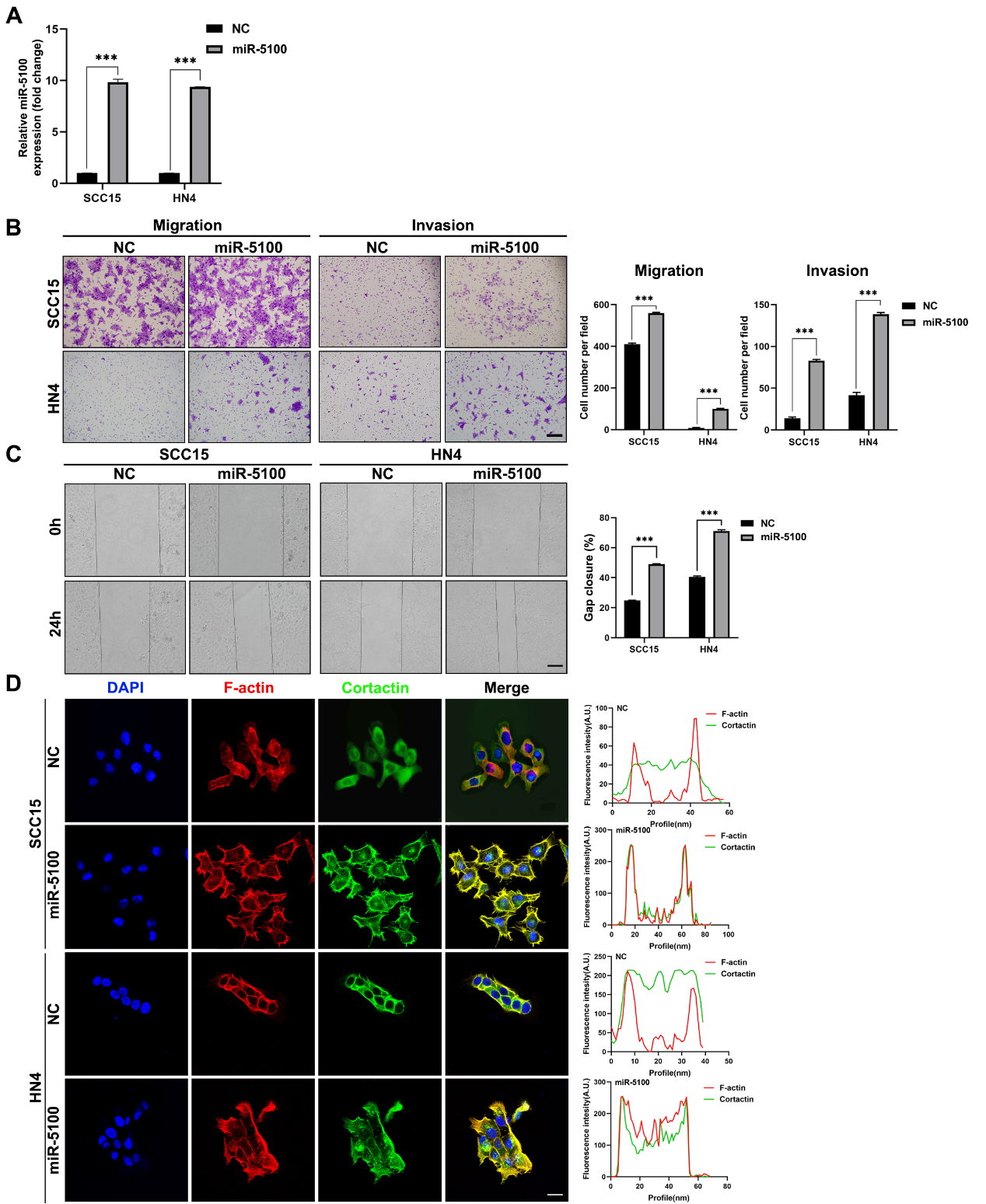
**

**Supplementary Fig. 3 MiR-5100 strengthens motility of HNSCC cells *in vitro*.** **A** The qPCR result of enforced expression of miR-5100 in SCC15 and HN4 cells. The level of miR-5100 in the transfected SCC15 and HN4 cells with miR-5100 mimic was analyzed 48 hours after transfection. **B and C** Increased expression of miR-5100 in SCC15 and HN4 cells promoted migration and invasion (**B**), and healing velocity (**C**) of these transfected cells. Scale bar in (**B**), 100 μm. Scale bar in (**C**), 200 μm. **D** Representative pictures of immunofluorescence of F-action and Cortaction in SCC15 and HN4 cells transfected with miR-5100 mimic and negative control oligo, respectively. Scale bar, 20 μm. Data in this figure, mean ± SD, ****P*<0.001.

**
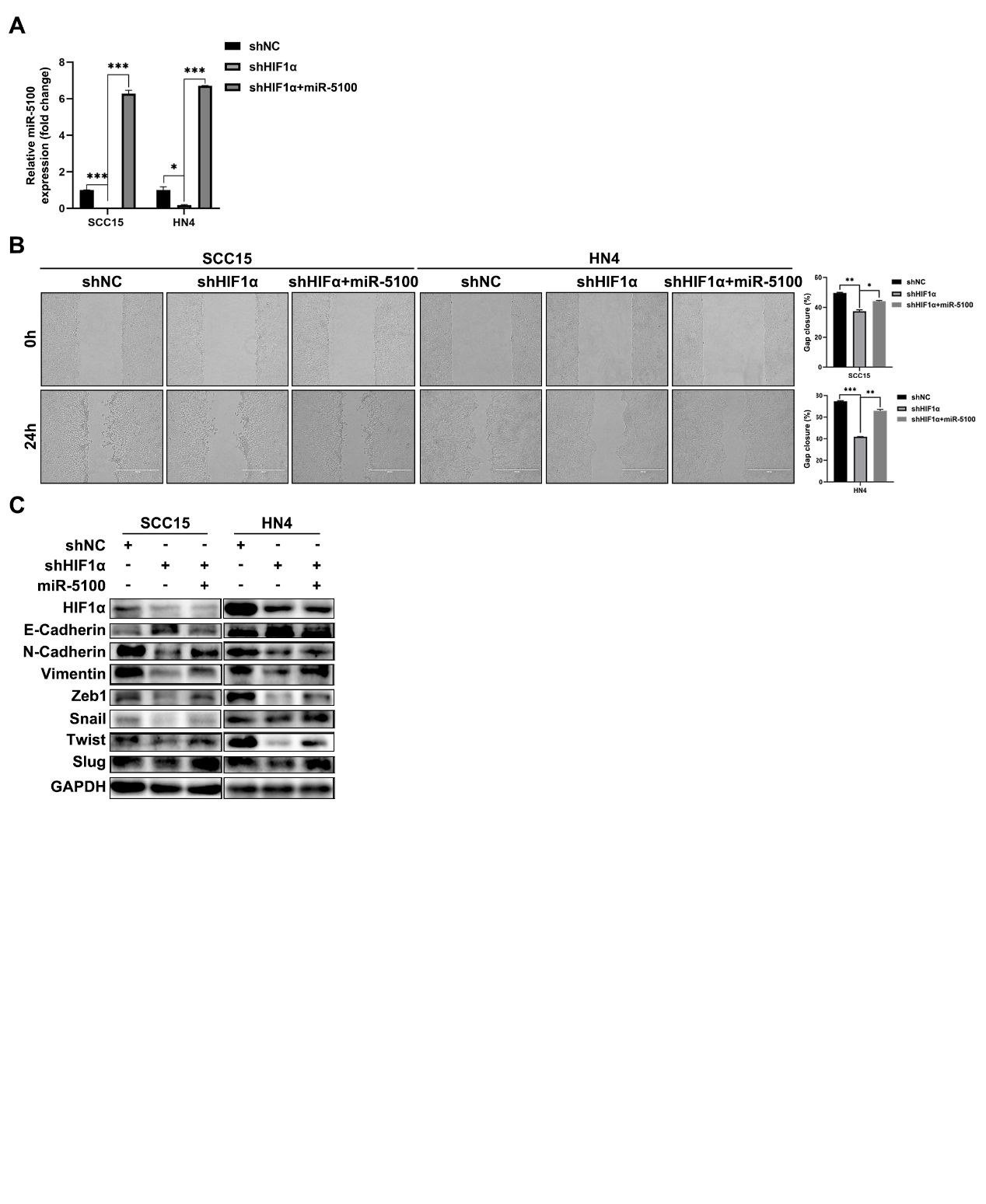
**

**Supplementary Fig. 4 The re-repression of miR-5100 improves motility ability of HIF1α-silenced HNSCC cells *in vitro*.** **A** The expression of miR-5100 was determined using qPCR in SCC15 and HN4 cells as indicated. **B** Enhanced expression of miR-5100 relieved the inhibitory effect of shHIF1α on the wound healing ability of SCC15 and HN4 cells. Scale bar, 400 μm. **C** The abundance of HIF1α and EMT-related markers was probed in indicated SCC15 and HN4 cells. Data in this figure, mean ± SD, **P*<0.05, ***P*<0.01, ****P*<0.001.


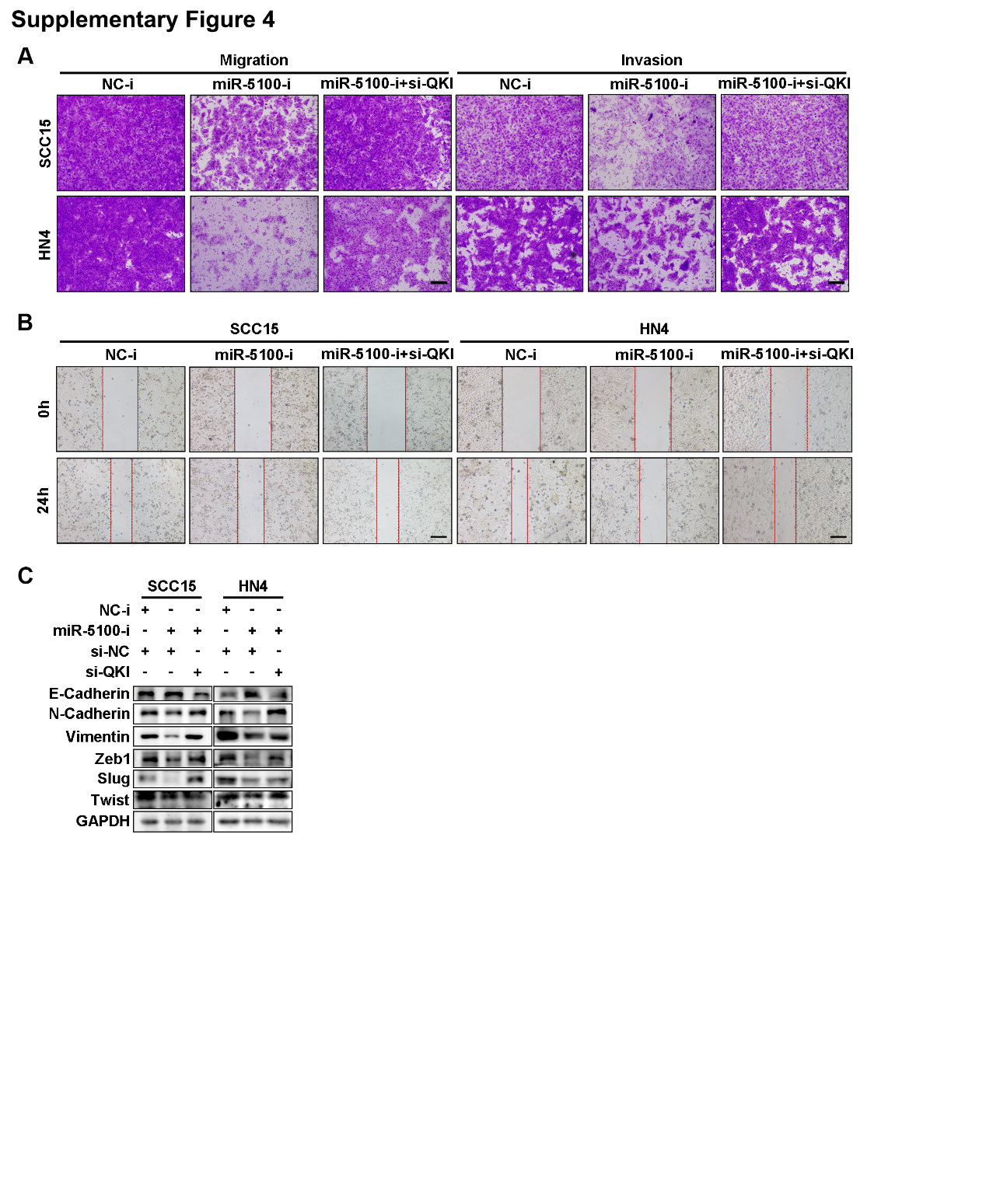


**Supplementary Fig. 5 The knockdown of QKI promotes motility capacity of miR-5100-reduced HNSCC cells *in vitro*.** **A** SCC15 and HN4 cells were transfected with control oligo, miR-5100 inhibitor, or miR-5100 plus siRNAs against QKI, respectively. Forty-eight hours after introduction, transwell assay was conducted to assess the capacities of migration and invasion of these transfected HNSCC cells. Scale bar, 100 μm. **B** The wound healing assay was performed in SCC15 and HN4 cells as described in (**A**). Scale bar, 200 μm. **C** The abundance of EMT-related markers was analyzed in indicated SCC15 and HN4 cells.


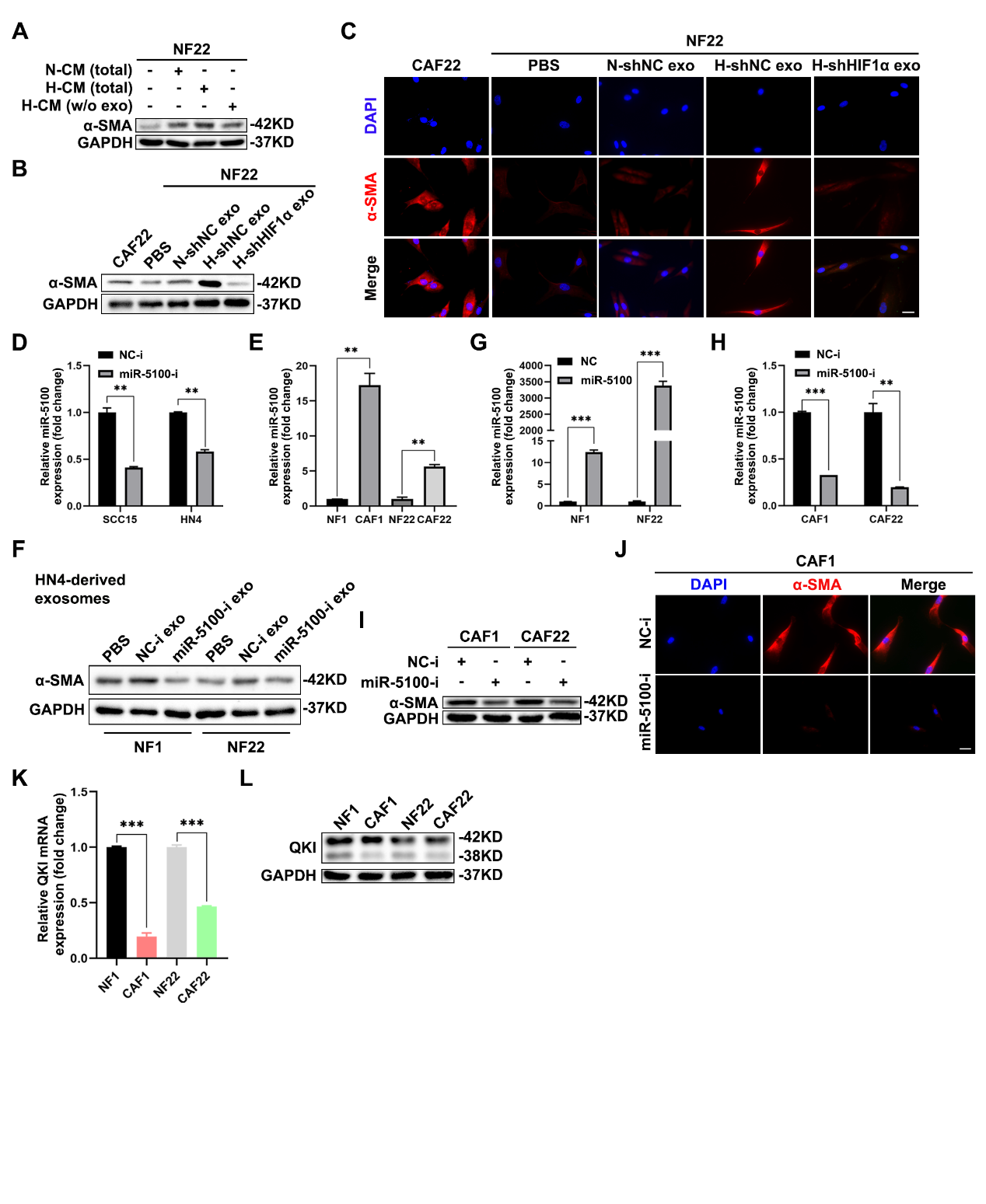


**Supplementary Fig. 6 Hypoxic HNSCC-derived exosomal miR-5100 induces conversion of NFs into CAFs.** **A** The abundance of α-SMA was analyzed in NF22 cells treated with indicated conditioned medium. N-CM (total), normoxic SCC15 cells-derived conditioned medium. H-CM (total), hypoxic SCC15 cells-derived conditioned medium. H-CM (w/o exo), hypoxic SCC15 cells-derived conditioned medium free from exosomes. **B and C** The level of α-SMA was detected using immunoblotting and immunofluorescence in CAF22 and NF22 cells treated with PBS or indicated exosomes. Scale bar in (**C**), 20 μm. **D** The qPCR result of reduced expression of miR-5100 in SCC15 and HN4 cells. **E** The result of qPCR assay identified a significant increase of miR-5100 in CAF1 and CAF22 relative to NF1 and NF22, respectively. **F** The abundance of α-SMA was detected using immunoblotting in NF1 and NF22 cells treated with exosomes from miR-5100-reduced HN4 cells. **G and H** The qPCR results of enforced and reduced expression of miR-5100 in NF1/NF22 cells transfected with miR-5100 mimic and CAF1/CAF22 cells transfected with miR-5100 inhibitor. **I and J** The level of α-SMA in CAF1 and CAF22 cells transfected with miR-5100 inhibitor was measured using immunoblotting (**I**) and immunofluorescence (**J**). Scale bar in (**J**), 20 μm. **K and L** Relative expression of QKI was detected using qPCR and immunoblotting in NFs as well as CAFs. Data in this figure, mean ± SD, ***P*<0.01, ****P*<0.001.
