# Supplementary material for: Hypoxia-induced miR-5100 promotes exosome-mediated activation of cancer-associated fibroblasts and metastasis of head and neck squamous cell carcinoma
Source: Cell Death Dis. 2024 Mar 14;15(3):215. doi: 10.1038/s41419-024-06587-9 (PMC10940661; doi:10.1038/s41419-024-06587-9)

F1M QKI

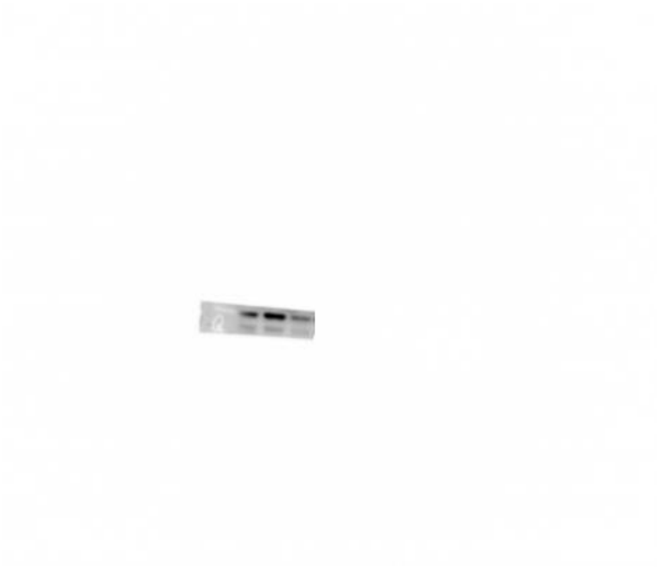

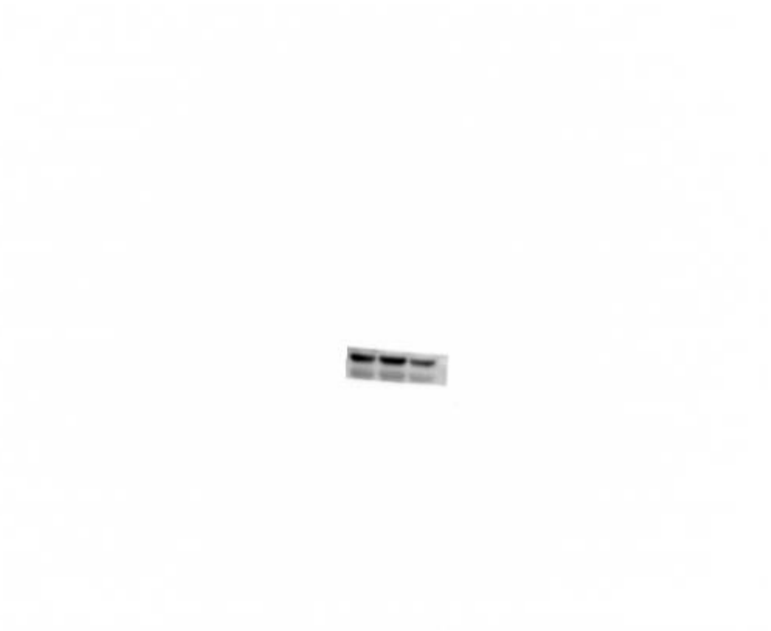

F1M GAPDH

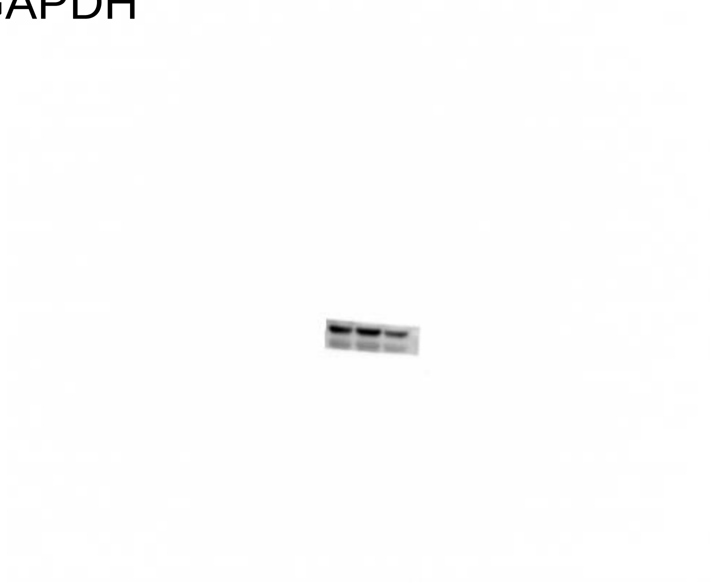

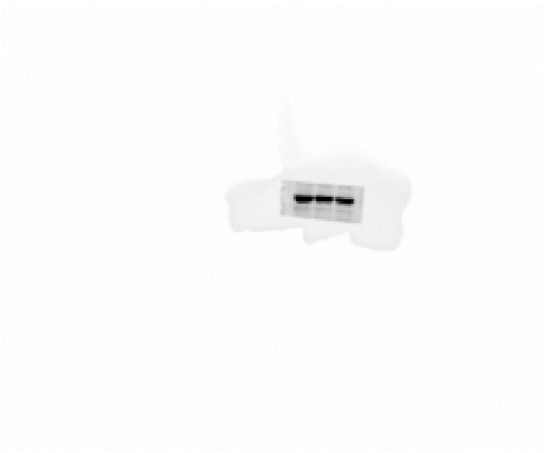

F3B α-SMA

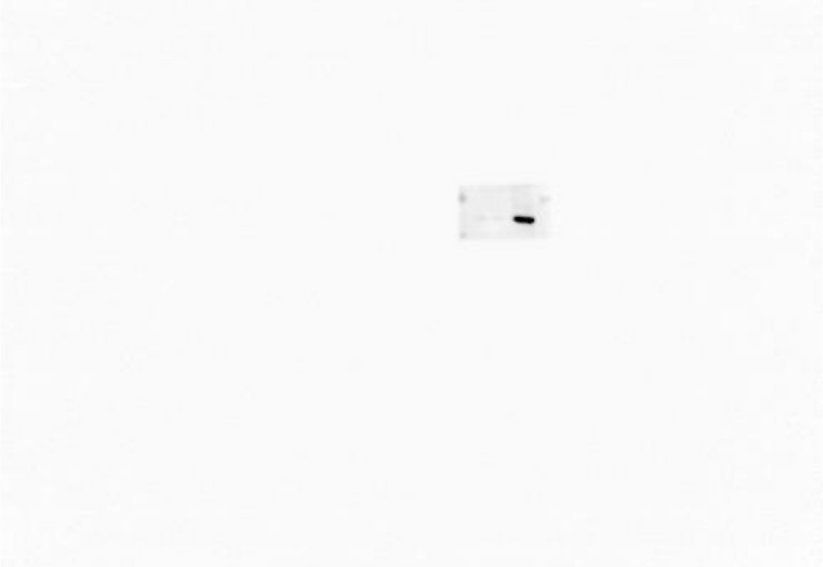

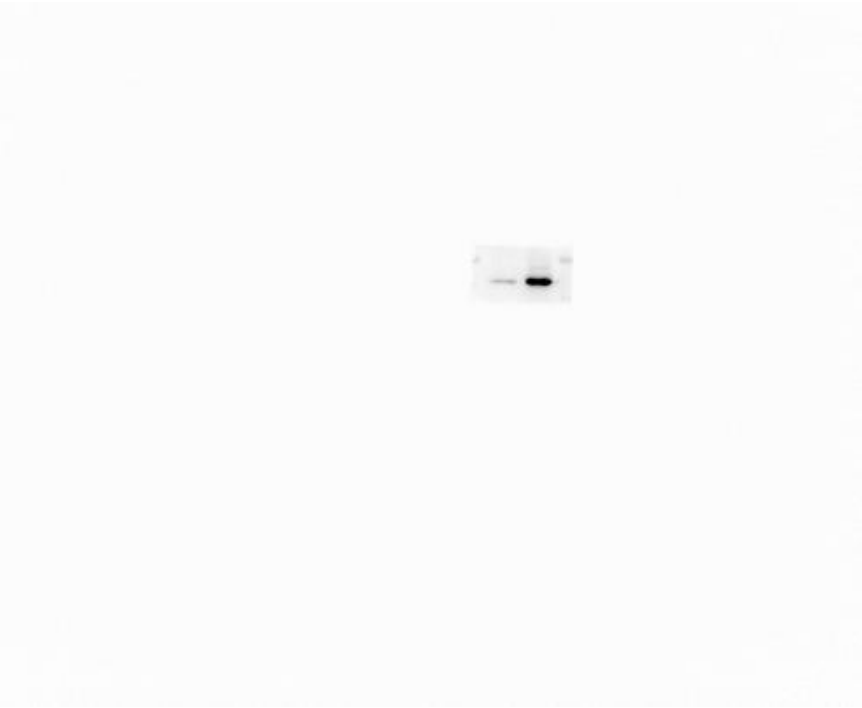

F3B GAPDH

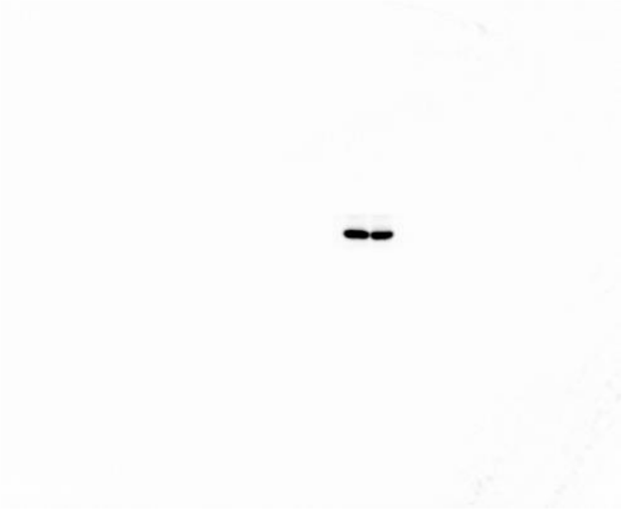

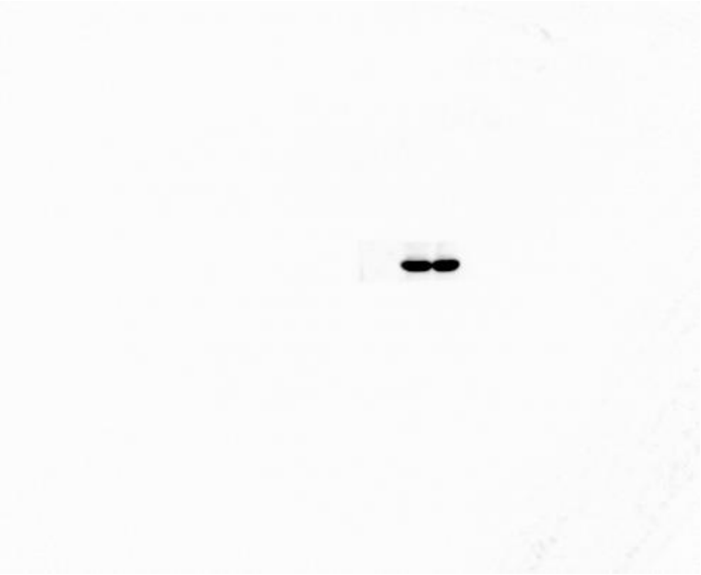

F3C α-SMA

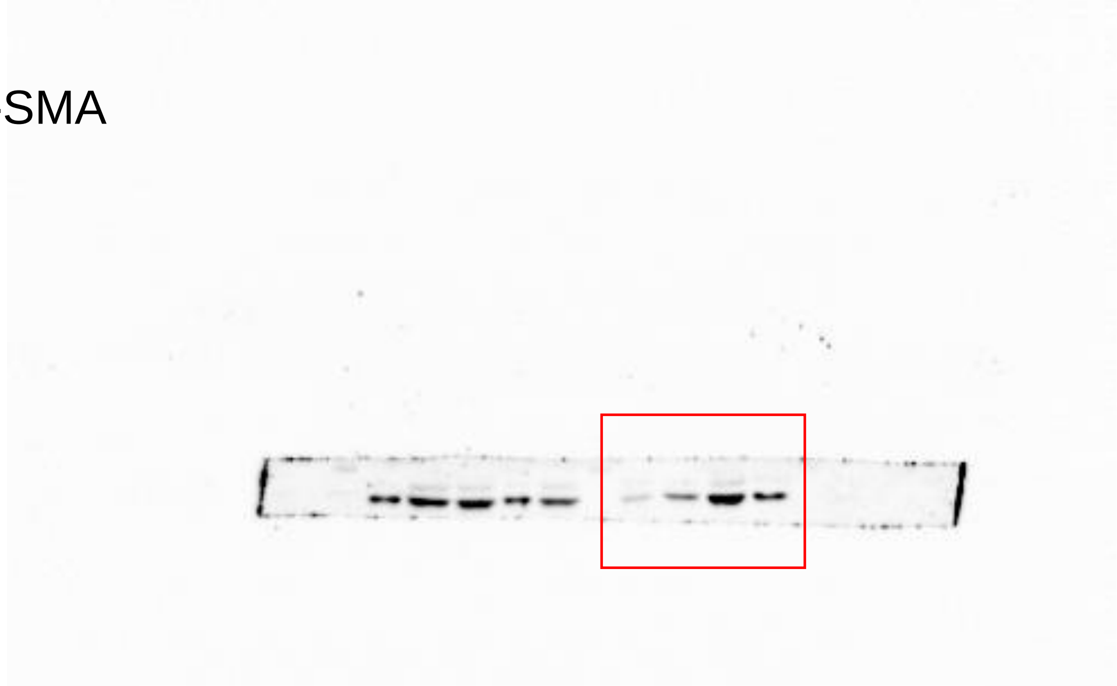

F3C GAPDH

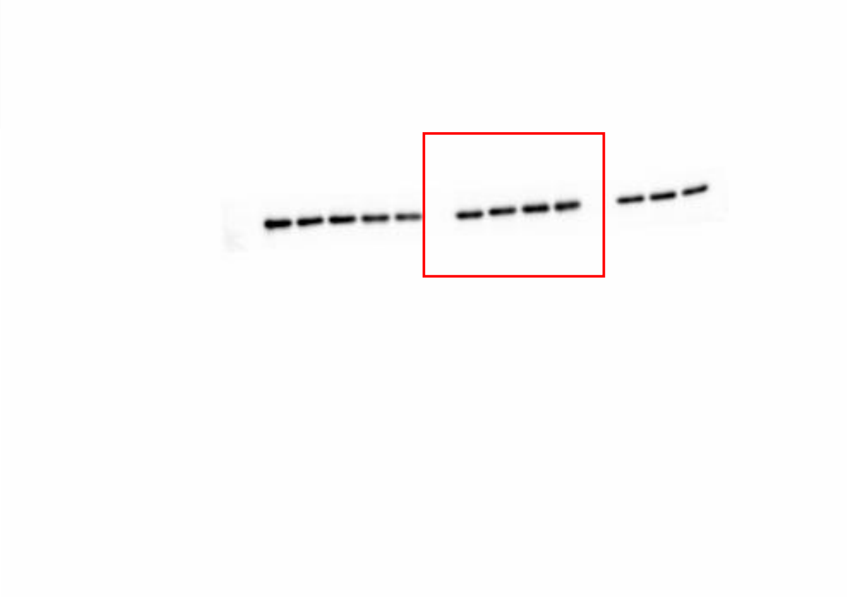

F3F ALIX

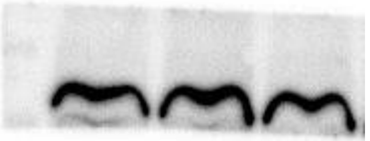

F3F CD9

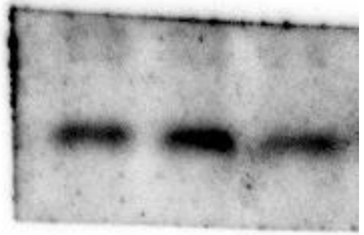

F3F CD63

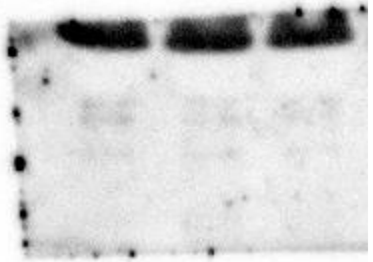

F3H α-SMA

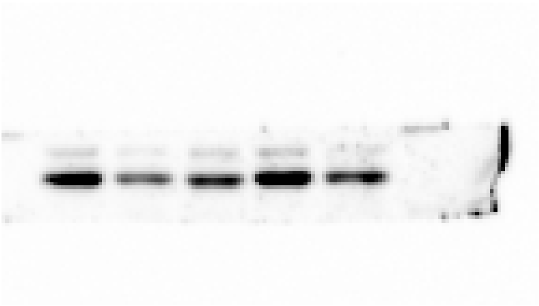

F3H GAPDH

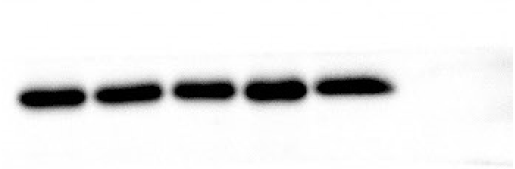

F4C α-SMA

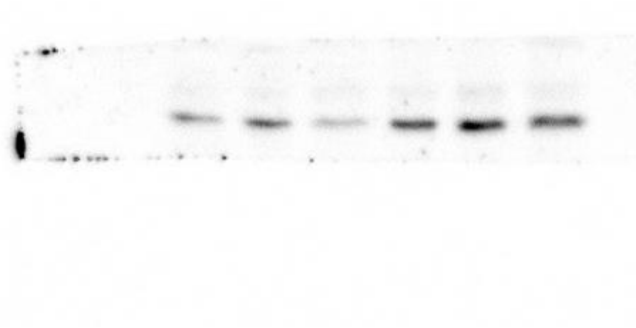

F4C GAPDH

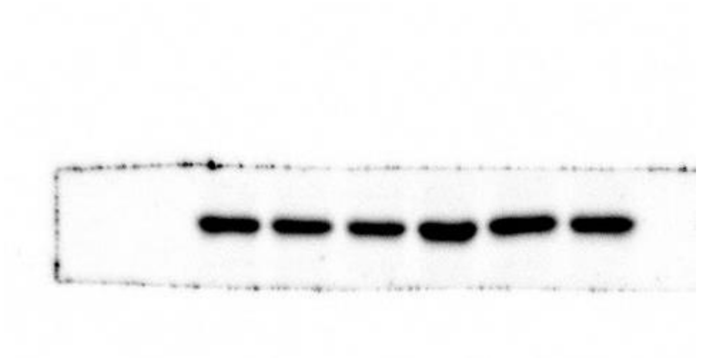

F4D α-SMA

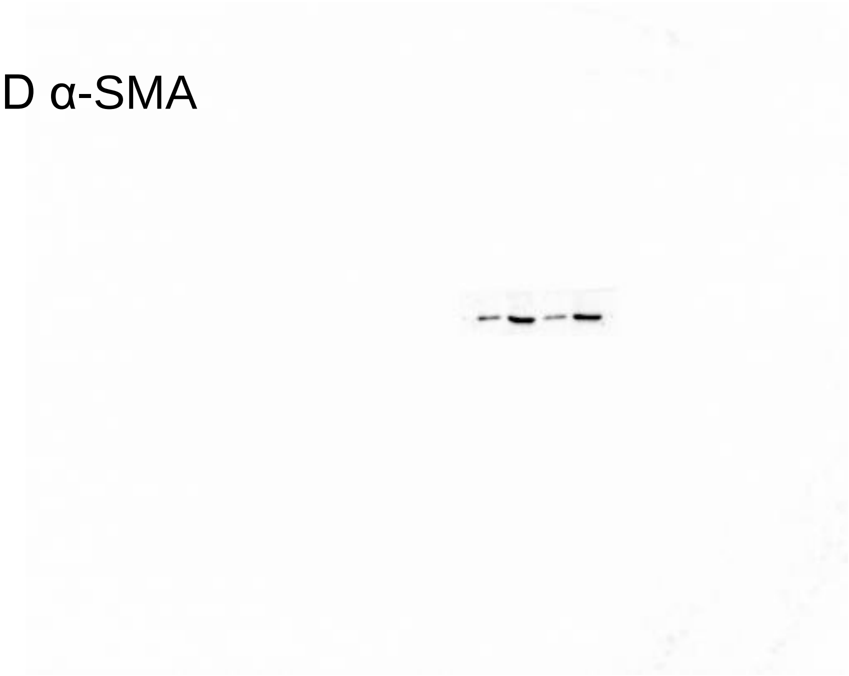

F4D GAPDH

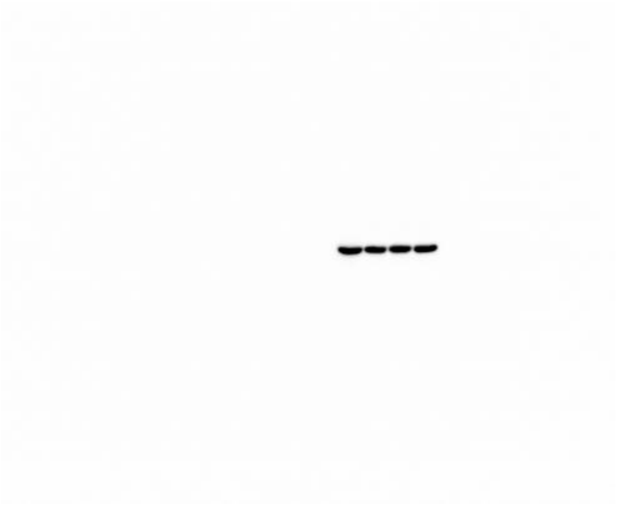

F4F QKI

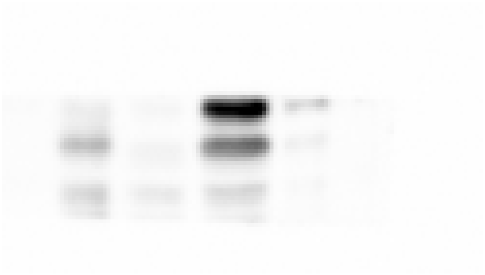

F4G QKI

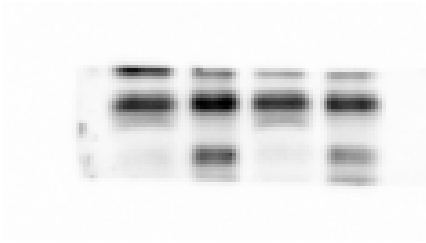

F4F GAPDH

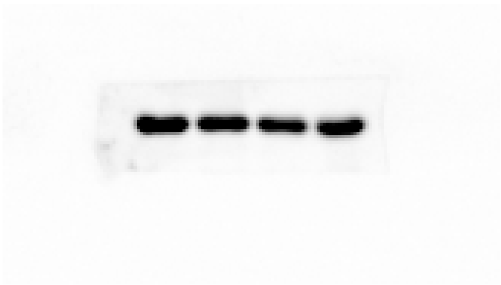

F4G GAPDH

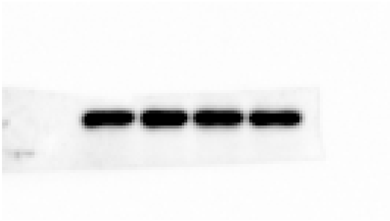

F4J QKI

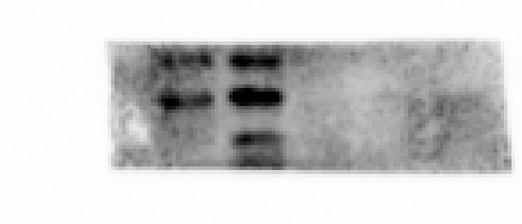

F4J p-AKT

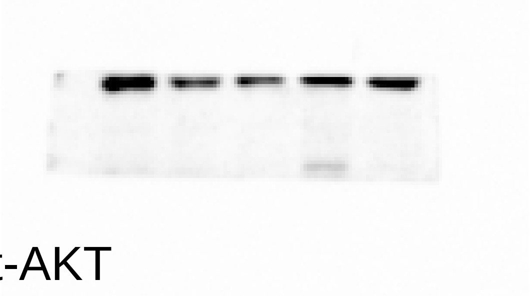

F4J t-AKT

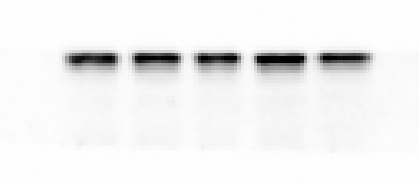

F4J p-STAT3

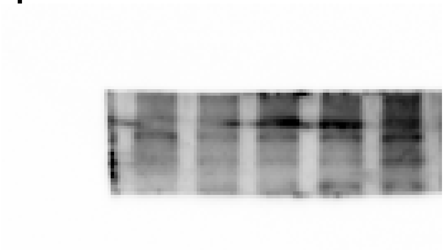

F4J t-STAT3

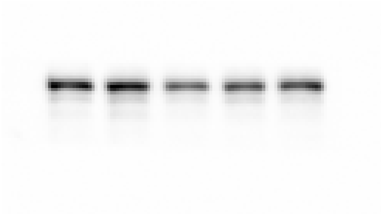

F4J α-SMA

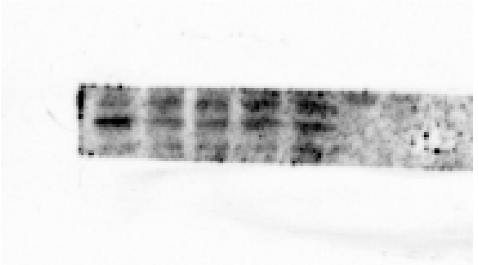

F4J GAPDH

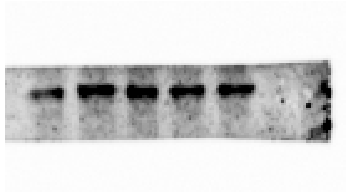

F4K α-SMA

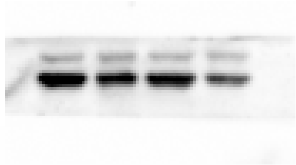

F4L α-SMA

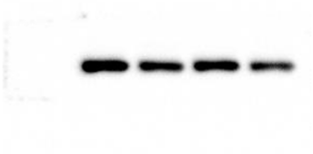

F4K GAPDH

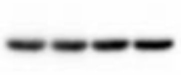

F4L GAPDH

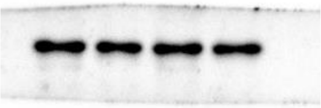

F5D E-Cad

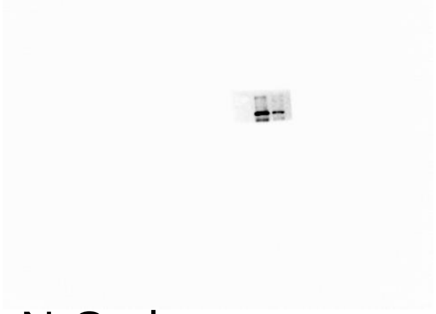

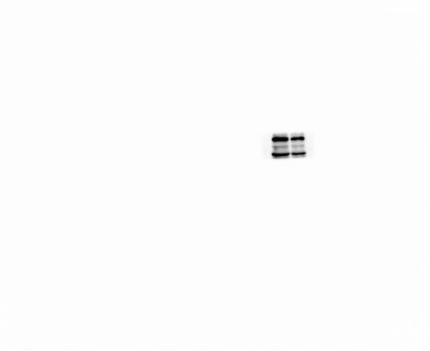

F5D N-Cad

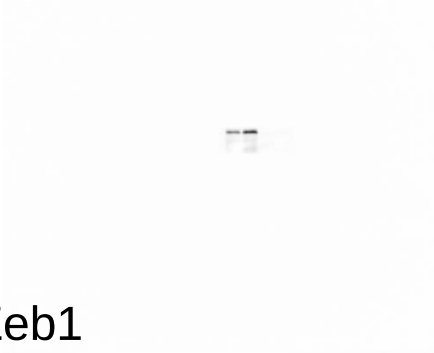

F5D Zeb1

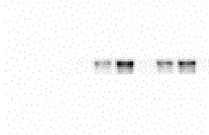

F5D Vim

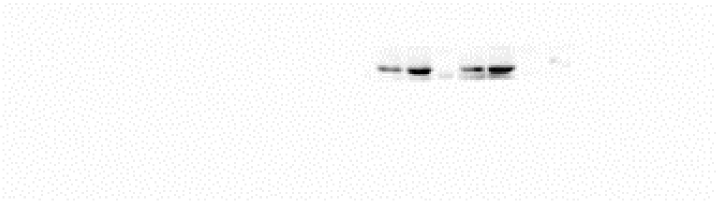

F5D Snail

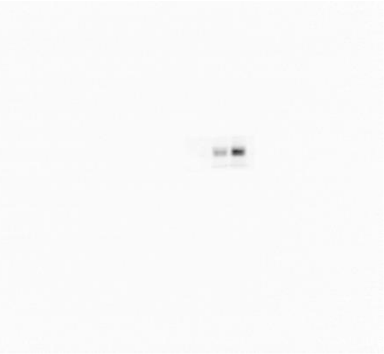

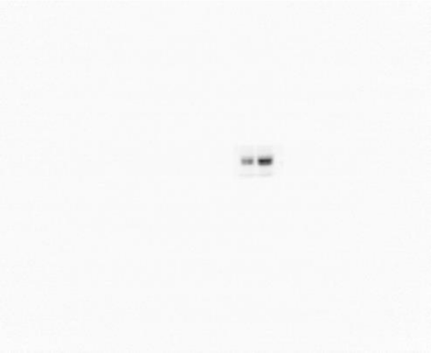

F5D Twist

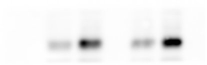

F5D Slug

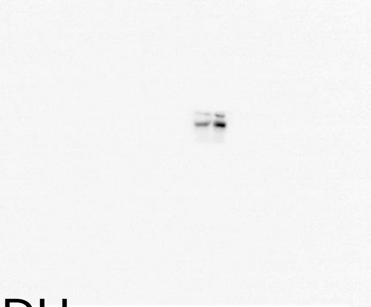

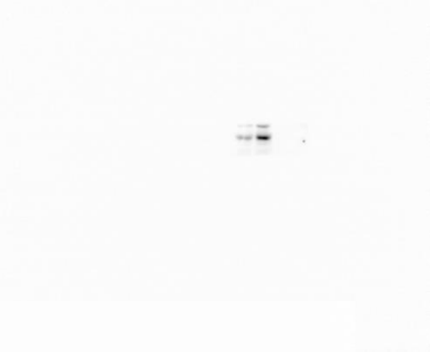

F5D GAPDH

SF2B HIF1A

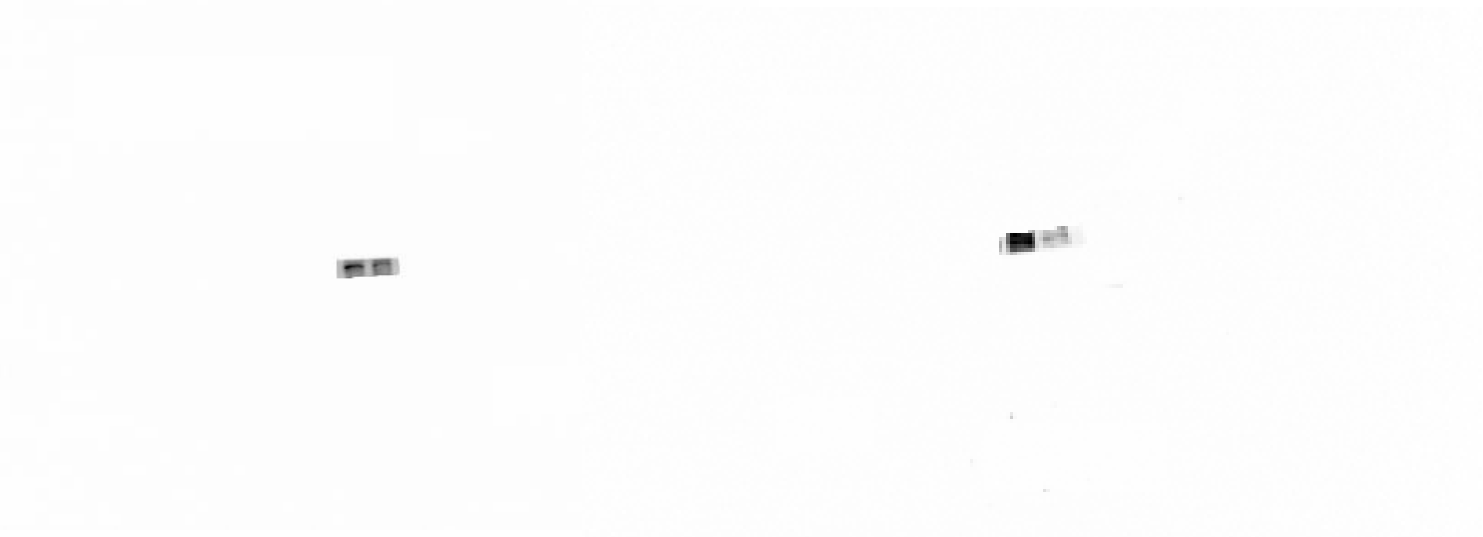

SF2B GAPDH

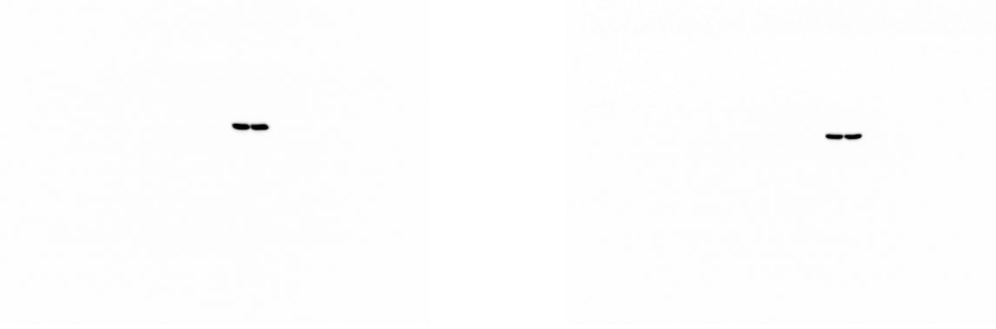

SF2E E-Cad

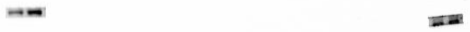

SF2E N-Cad

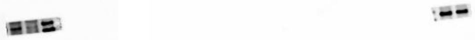

SF2E Vim

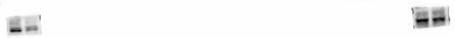

SF2E Zeb1

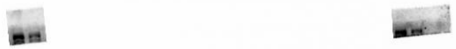

SF2E Snail (HN4+SCC15)

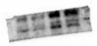

SF2E Slug

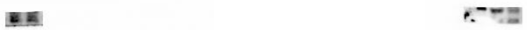

SF2E GAPDH

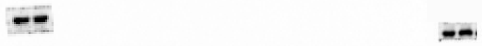

SF4C HIF1A

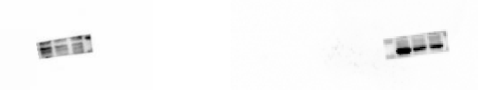

SF4C E-Cad

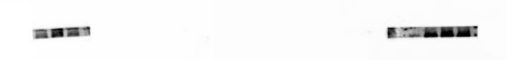

SF4C N-Cad

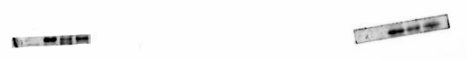

SF4C Vim

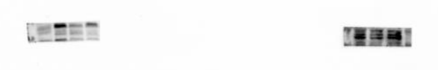

SF4C Zeb1

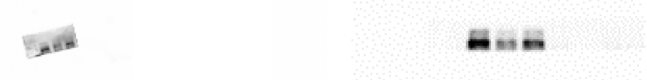

SF4C Snail

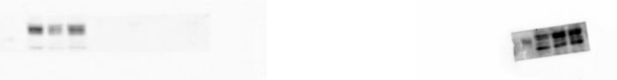

SF4C Twist

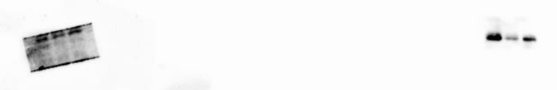

SF4C Slug

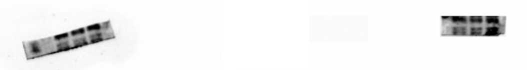

SF4C GAPDH

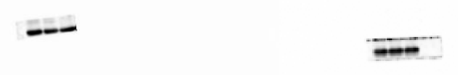

SF5C E-Cad

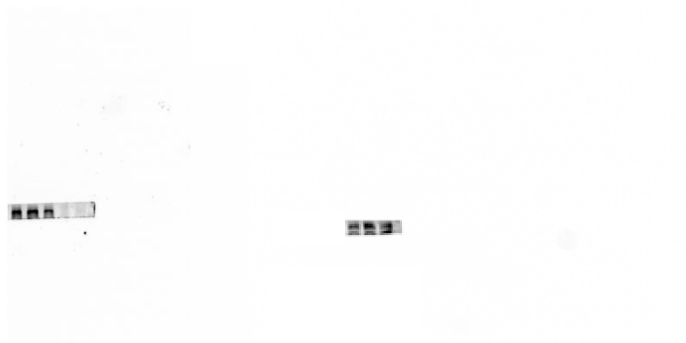

SF5C N-Cad

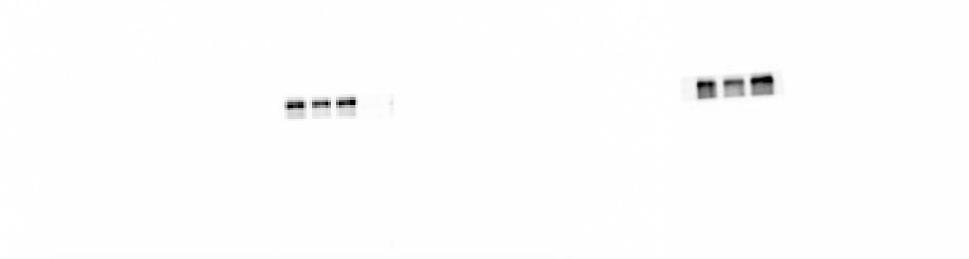

SF5C Vim

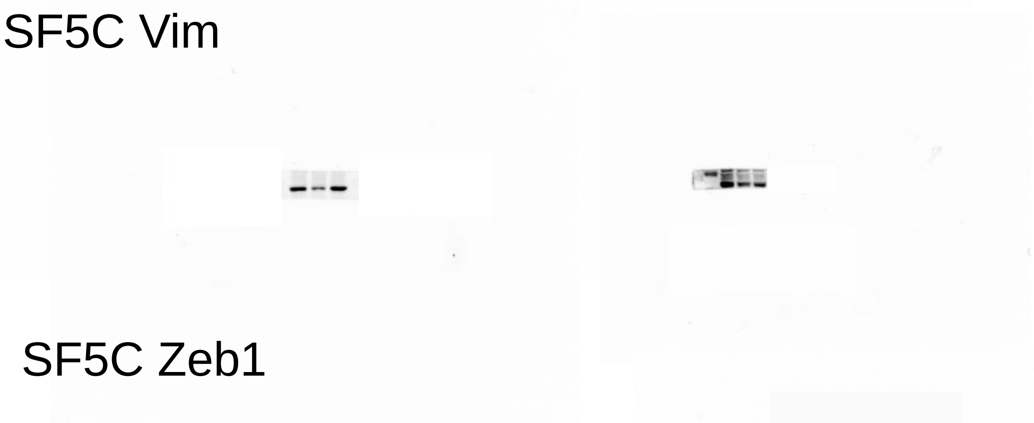

SF5C Zeb1

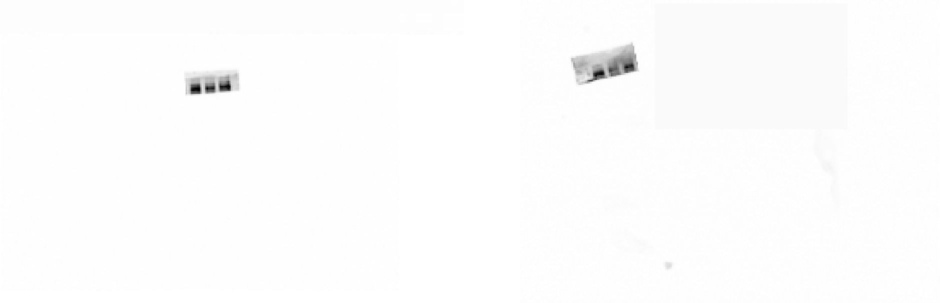

SF5C Slug

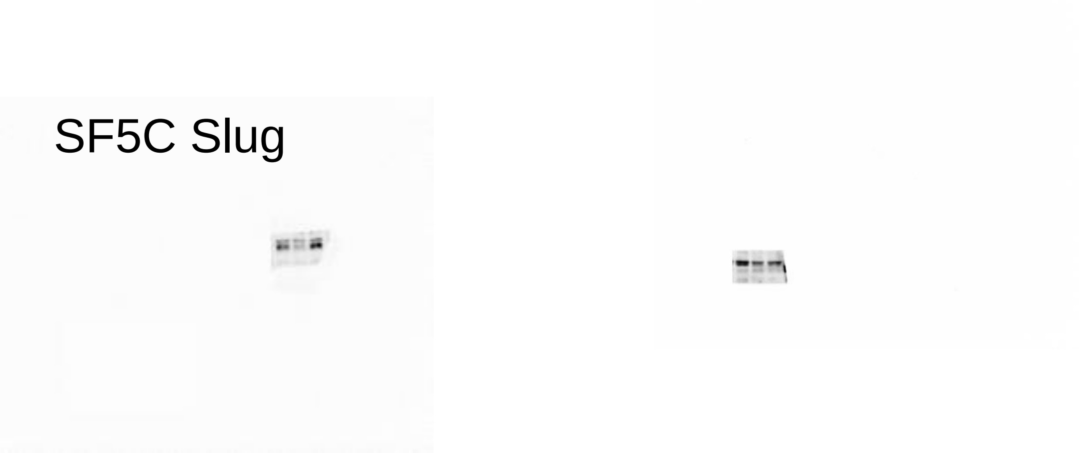

SF5C Twist

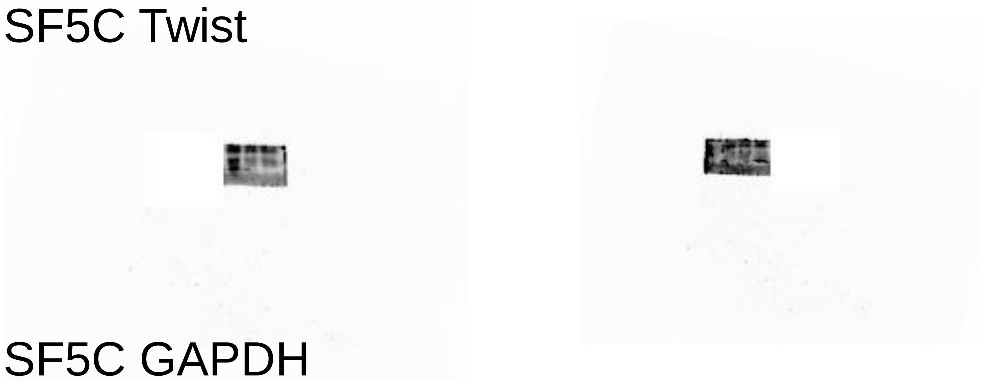

SF5C GAPDH

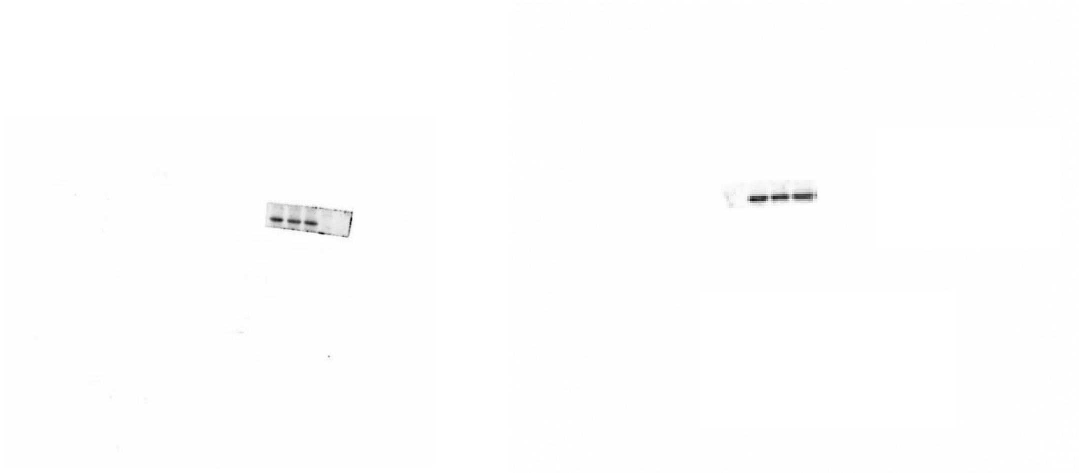

SF6A α-SMA

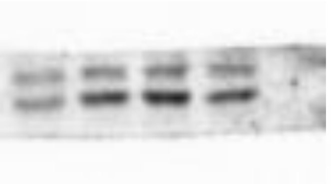

SF6B α-SMA

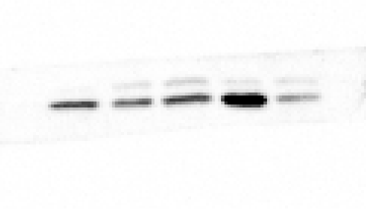

SF6A GAPDH

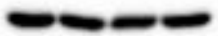

SF6B GAPDH

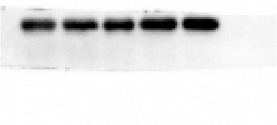

SF6F α-SMA

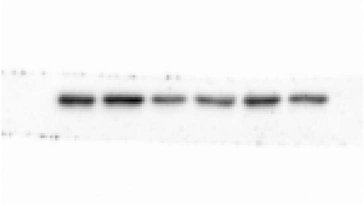

SF6F GAPDH

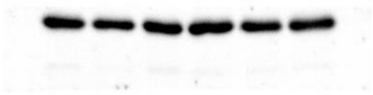

SF6I α-SMA

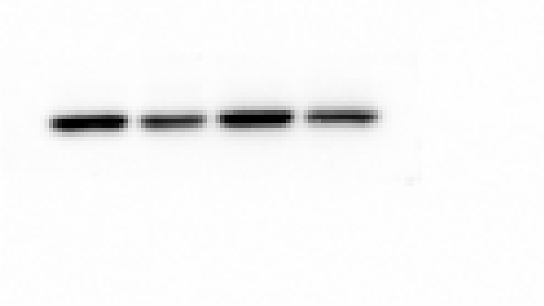

SF6L QKI

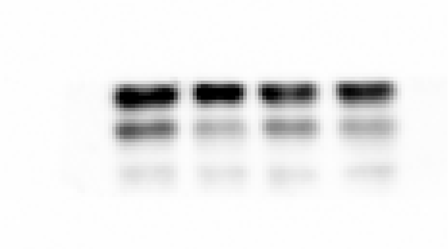

SF6I GAPDH

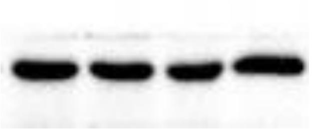

SF6L GAPDH

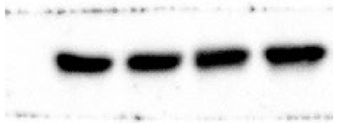

Supplement: Supplementary file 2 — Full uncut gels [file 41419_2024_6587_MOESM2_ESM.pdf]
